# A CAF01-adjuvanted whole asexual blood-stage liposomal malaria vaccine induces a CD4^+^ T-cell-dependent strain-transcending protective immunity in rodent models

**DOI:** 10.1128/mbio.02547-23

**Published:** 2023-11-14

**Authors:** Winter A. Okoth, Mei-Fong Ho, Mehfuz Zaman, Emily Cooper, Priyanka Som, Mark Burgess, Maddison Walton, Reshma J. Nevagi, Lynette Beattie, Declan Murphy, Danielle I. Stanisic, Michael F. Good

**Affiliations:** 1 Institute for Glycomics, Griffith University, Southport, Queensland, Australia; 2 The Peter Doherty Institute for Infection and Immunity, University of Melbourne, Melbourne, Victoria, Australia; NIAID/NIH, Rockville, Maryland, USA

**Keywords:** CAF01 adjuvanted, whole blood stage, malaria, vaccine, liposomes

## Abstract

Malaria is a leading cause of illness and death in children under 5 years of age in Sub-Saharan Africa. Currently, there is no highly efficacious malaria vaccine capable of inducing long-lasting immunity. This has increased interest in exploring different vaccine development strategies, including whole-parasite vaccines, and utilizing more effective adjuvant delivery systems. Here, we evaluate the immunogenicity and protective efficacy of a whole-parasite *Plasmodium yoelii* 17X blood-stage vaccine formulated with the clinically tested cationic adjuvant formulation, CAF01. The vaccine protected both inbred and outbred mice, and protected mice from homologous and heterologous challenge infections. Tracking of RBC subsets in vaccinated mice demonstrated that vaccine-induced immunity cleared parasitized normocytes as well as reticulocytes. Infection prior to vaccination led to an augmented level of protection and boosted immunity post vaccination. The vaccine induced parasite-specific IgG, mainly of the IgG1 subclass, and cellular responses, with a mixed Th1/Th2/Th17 cytokine profile. Mechanistic studies demonstrated that CD4^+^ T-cells (but not CD8^+^ T-cells), and Th1 and Th2 cytokines (interferon gamma and tumor necrosis factor, IL-10) were critical in controlling parasitemia and survival following challenge. Vaccinated µMT mice were not protected, suggesting that B-cells also play a role in protective immunity. Depletion of splenic macrophages with clodronate did not affect vaccine efficacy. These pre-clinical findings will inform the transition of this vaccine candidate into clinical trials.

**IMPORTANCE:**

Malaria is a devastating disease that has claimed many lives, especially children <5 years of age in Sub-Saharan Africa, as documented in World Malaria Reports by WHO. Even though vector control and chemoprevention tools have helped with elimination efforts in some, if not all, endemic areas, these efforts have been hampered by serious issues (including drug and insecticide resistance and disruption to social cohesion caused by the COVID-19 pandemic). Development of an effective malaria vaccine is the alternative preventative tool in the fight against malaria. Vaccines save millions of lives each year and have helped in elimination and/or eradication of global diseases. Development of a highly efficacious malaria vaccine that will ensure long-lasting protective immunity will be a “game-changing” prevention strategy to finally eradicate the disease. Such a vaccine will need to counteract the significant obstacles that have been hampering subunit vaccine development to date, including antigenic polymorphism, sub-optimal immunogenicity, and waning vaccine efficacy.

## INTRODUCTION

Malaria remains a leading cause of illness and death among children ≤5 years in Sub-Saharan Africa. In 2021, the World Health Organization reported an estimated 247 million malaria cases and 619,000 malaria deaths globally (1). Concerted efforts to control malaria (e.g., interventions to reduce malaria prevalence and public-private sector collaboration) have resulted in some gains, including “malaria-free” certification of previously endemic countries (2) and the recent approval of the world’s first licensed malaria vaccine, Mosquirix, for use in African children at risk of *Plasmodium falciparum* infection (3). However, these gains are threatened by multiple factors, including the re-establishment of malaria in countries that had previously achieved elimination (4–6), the limited efficacy of Mosquirix (7), the disruption of social cohesion due to the COVID-19 pandemic (8), increasing prevalence of resistance to artemisinin (9) and insecticides (10), and the spread of the highly competent peri-urban vector, *Anopheles stephensi* to Africa (11).

Mosquirix (RTS, S/AS01) is a subunit vaccine targeting the liver stage of the parasite. In phase III clinical trials, its efficacy was <40% in 5–17-month-old children over a 4-year follow-up period, and protection was short lived, suggesting the need for frequent boosters to maintain protective immunity (12). Subunit vaccine candidates targeting the blood stage of the parasite have also shown zero-to-minimal protective efficacy when tested in the field (12–14). Factors such as antigenic polymorphism, immunological non-responsiveness, and sub-optimal adjuvants that are unable to induce and maintain sufficiently high antibody responses have all contributed to the poor efficacy of subunit vaccines. This has ignited interest in exploring the whole-parasite vaccine approach that will include a broader antigen repertoire (15–17).

Whole-parasite blood-stage vaccine candidates have been evaluated in rodent models using killed, genetically attenuated, or chemically attenuated parasites (15, 18–25). However, compared with whole-sporozoite malaria vaccines (17, 26, 27), there has been limited clinical assessment of the whole blood-stage parasite vaccine approach. We have shown that a single dose of *in vitro* chemically attenuated blood-stage *Plasmodium falciparum* parasites elicited strain and species-transcending parasite-specific cell-mediated immune responses in volunteers (28). However, effective cryopreservation of a whole-parasite blood-stage vaccine candidate that relies on intact red blood cells for vaccine efficacy (22) is a major challenge. We previously reported the efficacy of a cryopreserved whole-parasite blood-stage vaccine comprising killed, whole blood-stage *P. yoelii* 17X parasites formulated with liposomes, the toll-like receptor-4 agonist, 3D(6-acyl) PHAD (synthetic analog of monophosphoryl lipid A), and mannose (16). This proof-of-concept vaccine study (16) both confirmed the utility of liposomes as a delivery vehicle for killed whole blood-stage parasites and the maintenance of potency for a cryopreserved vaccine. However, this liposome platform technology has not been tested in any human studies (16).

Here, we further evaluate the use of liposomes as an adjuvant delivery system for a whole-parasite blood-stage vaccine using the clinically tested cationic liposomal adjuvant formulation, dimethyldioctadecylammonium (DDA)/trehalose 6,6′-dibehenate (TDB) (prepared in-house as DDA/TDB or obtained from Staten Serum Institute [SSI] as CAF01), with the rodent malaria model, *P. yoelii* 17X. CAF01 consists of the cationic surfactant, DDA, stabilized by a synthetic mycobacterial glycolipid, TDB (29). It has been shown to induce strong cellular and antibody responses (29, 30). Furthermore, it has an acceptable safety profile in humans and has been evaluated in numerous clinical studies in the context of subunit vaccines for tuberculosis, chlamydia, and malaria (31–34). Here, we assess the immunogenicity, protective efficacy, and mechanisms of vaccine-induced protective immunity induced by the vaccine and analyze the interaction between vaccination and malaria infection in boosting immunity.

## RESULTS

### Protective efficacy of the *Py*17X vaccine

**Fig 1:**
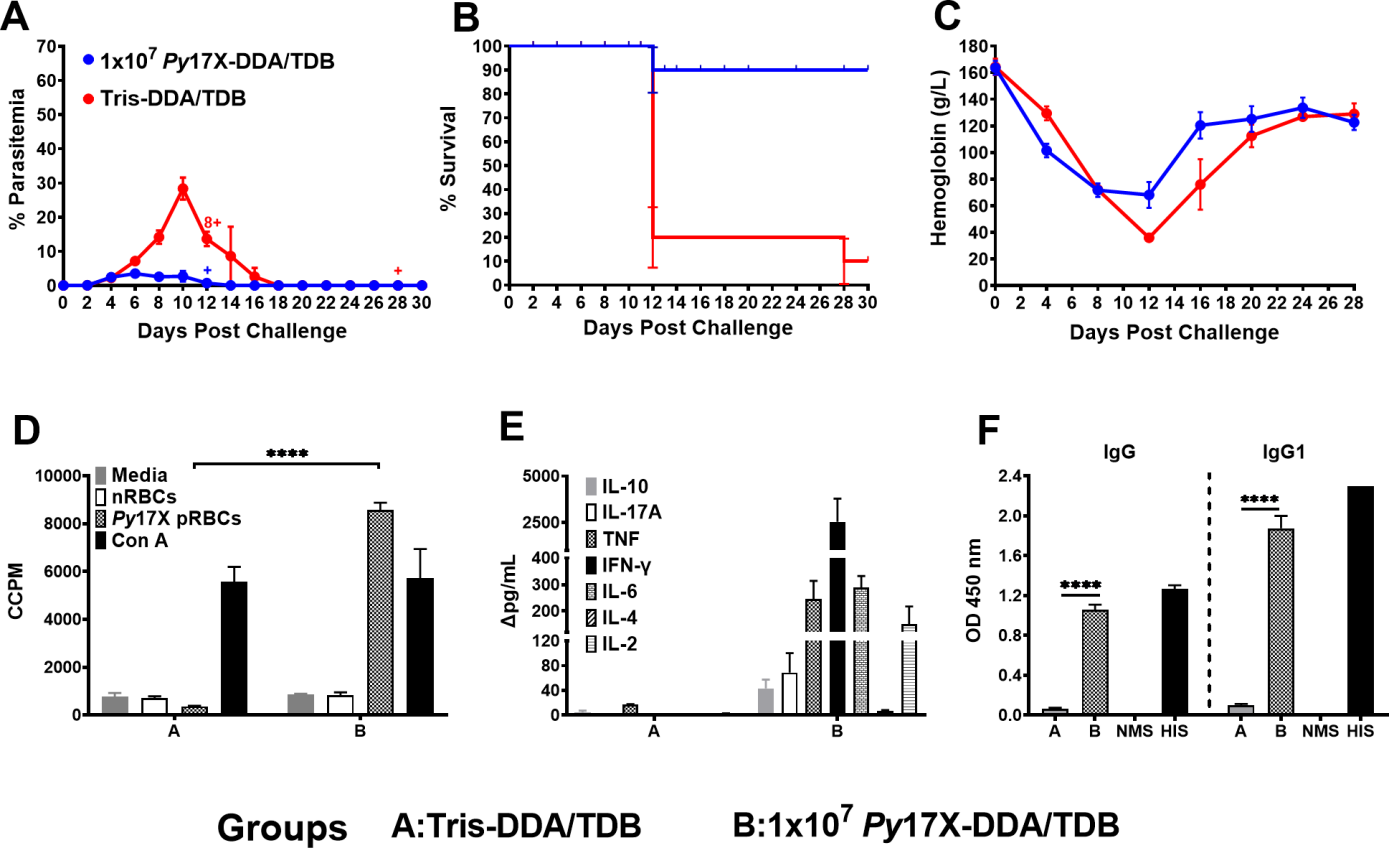
*Plasmodium yoelii* 17X (*Py*17X)-DDA/TDB whole blood-stage vaccine provides good protective efficacy in genetically outbred Swiss mice. Following three vaccinations in female Swiss mice (*n* = 10/group) with 1 × 10^7^ *Py*17X-DDA/TDB or Tris-DDA/TDB, mice were challenged intravenously (i.v.) with 1 × 10^5^ *Py*17X pRBCs after 4 weeks of rest. Monitoring (**A**) parasitemia by microscopy every 2 days, (**B**) Kaplan-Meier survival curve analysis plotted as a percent of survival, error bars show standard error of survival probability at each timepoint, (**C**) hemoglobin (g/L) every 4 days was recorded up to 30 days following challenge, (**D**) parasite-specific splenocyte proliferative responses of vaccinated and control mice groups assessed pre-challenge (*n* = 3 per group). Mouse spleen cells were incubated with different stimulants (media, nRBCs, pRBCs, and concanavalin A) in triplicates, and proliferation was estimated through incorporation of [^3^H]thymidine and represented as corrected counts per minute (CCPM). (**E**) Parasite-specific cytokine response was assessed using mouse Th1/Th2/Th17 cytokine bead array analysis of the culture supernatants of the splenocyte proliferation assay collected after 54 hours and represented as Δpg/mL (nRBCs background subtracted from pRBCs). (**F**) Parasite-specific IgG and IgG1 responses in vaccinated and control mice groups were measured by enzyme-linked immunosorbent assay using crude whole *Py*17X parasite antigen. Pre-challenge sera were tested in duplicate at 1:100 dilution. Normal mice sera (NMS) and hyperimmune mice sera (HIS) were included as negative and positive controls, respectively. Antibody response was presented as optical density (OD) measured at 450 nm wavelength. All data are expressed as means ± SEM. Cross sign represents euthanized mice. ****P < 0.0001.

To be a viable vaccine candidate to protect against malaria, a *P. falciparum* vaccine would need to protect individuals of diverse genetic backgrounds. We, thus, initially asked whether a *P. yoelii* 17X vaccine, formulated with DDA/TDB and delivered subcutaneously (s.c.), could protect an outbred population of mice. Swiss mice (13 per group) were given three doses of vaccine, each containing 10^7^ parasite equivalents, or three doses of Tris-DDA/TDB (control group). Three mice in each group were sacrificed for immunological assays, and the remaining 10 were challenged 1 month later by an intravenous infusion of 10^5^ parasitized red blood cells (pRBCs). We observed that vaccinated mice had a significantly lower mean peak parasitemia (3.50% ± 0.65%,) compared to control mice (28.4% ± 3.22%) (*P* = 0.0002, area under the curve [AUC] analysis) and significantly enhanced survival (90% vs 10%; *P* = 0.0013, log-rank test analysis) (Fig. 1A and B). While both vaccinated and control mice suffered a drop in hemoglobin levels, this was less in the vaccinated mice (Fig. 1C). There was an induction of parasite-specific splenocyte proliferative responses, mixed Th1/Th2/Th17 cytokine responses, and antibody responses (predominantly IgG1) in vaccinated mice (Fig. 1D, E, and F). Having established the viability of the model, we turned to inbred mice, where there would be less animal-to-animal variation, to study vaccine optimization, the role of the adjuvant, and to analyze the immune responses to the vaccine.

### Vaccine optimization

**Fig 2:**
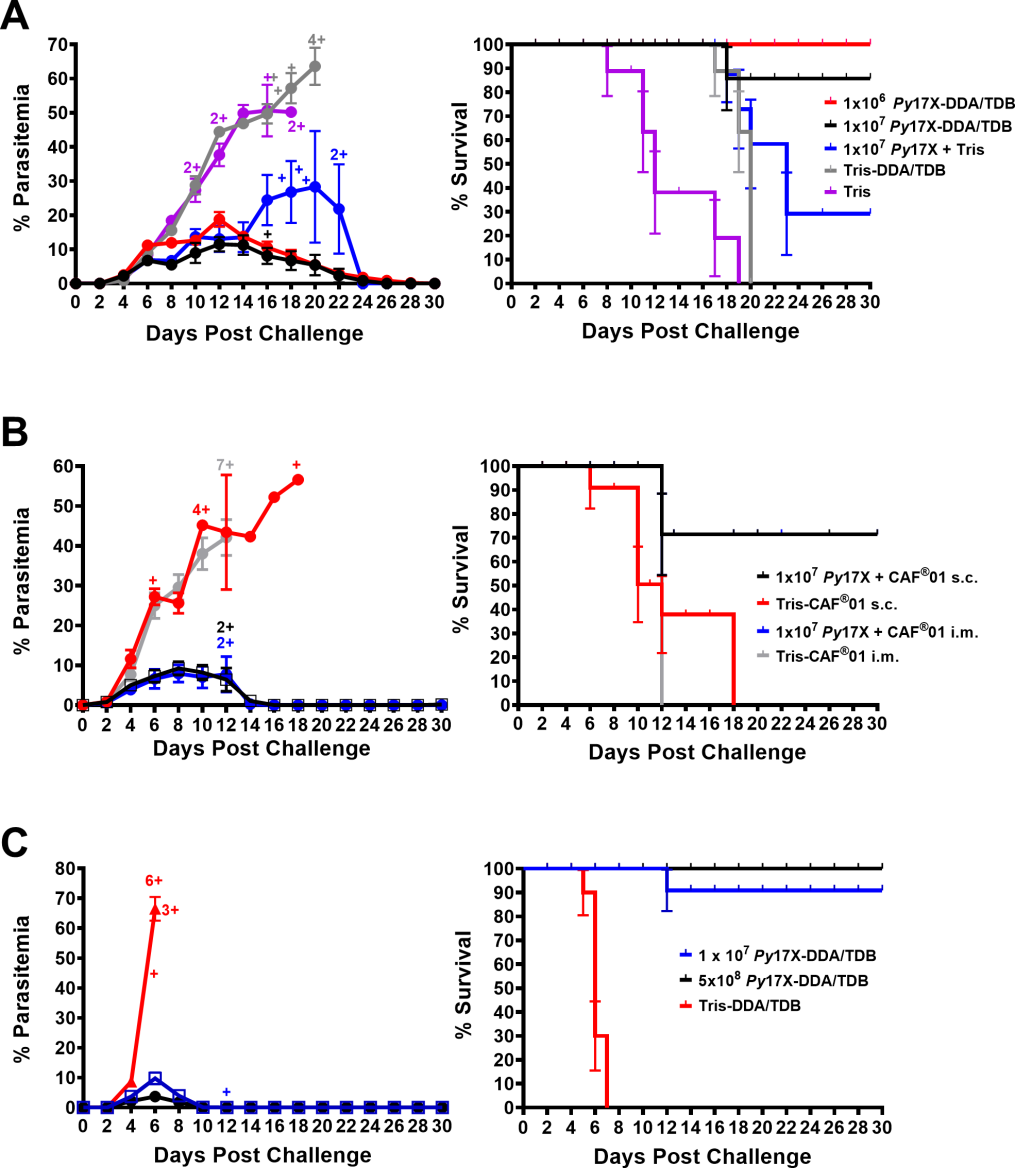
*Py*17X/CAF01 whole blood-stage vaccine provides good protective efficacy against homologous and cross-strain heterologous blood-stage challenge in inbred mice. (**A**) Dose-ranging study to determine optimal parasite antigen dose required to induce protective immunity following vaccination with a *P. yoelii* 17X-DDA/TDB vaccine in female BALB/c mice. Groups of BALB/c mice (*n* = 10/group) were immunized with three doses of 10^6^ or 10^7^ *Py*17X-DDA/TDB vaccine, 10^7^ *Py*17X in Tris, Tris-DDA/TDB, or Tris. (**B**) Route of administration: protective efficacy of a *P. yoelii* 17X/CAF01 vaccine administered via s.c. or intramuscularly (i.m.) routes in female BALB/c mice against homologous challenge. Groups of BALB/c mice (*n* = 10/group) were immunized with three doses of 10^7^*Py*17X/CAF01 vaccine or Tris-CAF01 control administered s.c. or i.m. (**C**) Cross-strain heterologous protection in female BALB/c mice. Groups of BALB/c mice (*n* = 10/group) were immunized with three doses of 10^7^ or 5 × 10^8^ *Py*17X-DDA/TDB vaccine or Tris-DDA/TDB. In all studies, mice were rested for 4 weeks following second booster dose and received homologous (**A and B**) or heterologous (**C**) challenge with 10^5^ *Py*17X or *Plasmodium yoelii* YM pRBCs intravenously, respectively. Parasitemia and Kaplan-Meier survival curve analysis plotted as a percent of survival, error bars show standard error of survival probability at each timepoint, were recorded up to 30 days following challenge. All data are expressed as means ± SEM. Cross sign represents euthanized mice.

A dose ranging study was undertaken in BALB/c mice (Fig. 2A). Female mice (10 per group) were immunized with vaccines containing the equivalent of 10^6^ or 10^7^ *Plasmodium yoelii* 17X (*Py*17X) pRBCs with DDA/TDB, per dose; control mice received 10^7^ *Py*17X in Tris, Tris-DDA/TDB, or Tris alone. Mice received three doses of vaccine or control preparations each 2 weeks apart. We again observed that vaccinated mice were protected against homologous challenge infection and that the higher dose of vaccine was slightly more effective in reducing peak parasitemia (11.5% ± 2.1% vs 18.8% ± 2.2%; *P* > 0.05 AUC). A range of 90%–100% of vaccinated mice survived the challenge. The control groups demonstrated that the DDA/TDB component of the vaccine was critical in controlling parasitemia and enhancing survival. Interestingly, mice given adjuvant in buffer without parasites (Tris-DDA/TDB) had prolonged survival (~2 days longer) compared to mice that just received buffer; however, Tris-DDA/TDB alone had no ability to control parasite burden (Fig. 2A). These experiments thus established the critical importance of both killed parasites and DDA/TDB cationic liposomes in the vaccine.

Given that we previously showed that a liposomal-based vaccine containing the toll-like receptor 4 agonist, PHAD, was effective (16), we assessed the effect of adding PHAD to the vaccine. However, following challenge, parasite control and survival were similar in mice immunized with vaccine formulated with or without PHAD (see Fig. S1). Interestingly, mice immunized with PHAD given alone as an adjuvant and mixed with the killed parasites showed intermediate levels of protection in terms of parasitemia and survival.

All previous experiments were conducted using an s.c. route of administration. We compared vaccine efficacy in mice administered the vaccine via the s.c. and intramuscular (i.m.) routes. The intranasal (i.n.) route was also tested as a previous vaccine study showed efficacy for a subunit malaria vaccine when administered via this route (35). However, all mice vaccinated via i.n. route succumbed to a challenge infection (data not shown). The s.c. and i.m. routes of immunization gave equivalent protection (Fig. 2B), and the s.c. route was routinely used for further experiments.

We next examined protective immunity to a heterologous strain of *P. yoelii*. Anticipating that cross-strain protection might be lower, we chose to vaccinate mice with either 10^7^ or 5 × 10^8^ *P. yoelii* 17X pRBCs formulated with DDA/TDB. Mice were intravenously (i.v.) challenged 1 month after the third dose of vaccine with 10^5^ *Plasmodium yoelii* YM pRBCs. We observed excellent control of parasitemia and survival following heterologous challenge and surprisingly found that peak parasitemias and survival were similar for both the standard and high doses of vaccine, although mice that received the higher vaccine dose demonstrated slightly reduced peak parasitemia compared to mice receiving the standard dose (Fig. 2C).

### Longevity and reproducibility of *Py*17X vaccine efficacy

**Fig 3:**
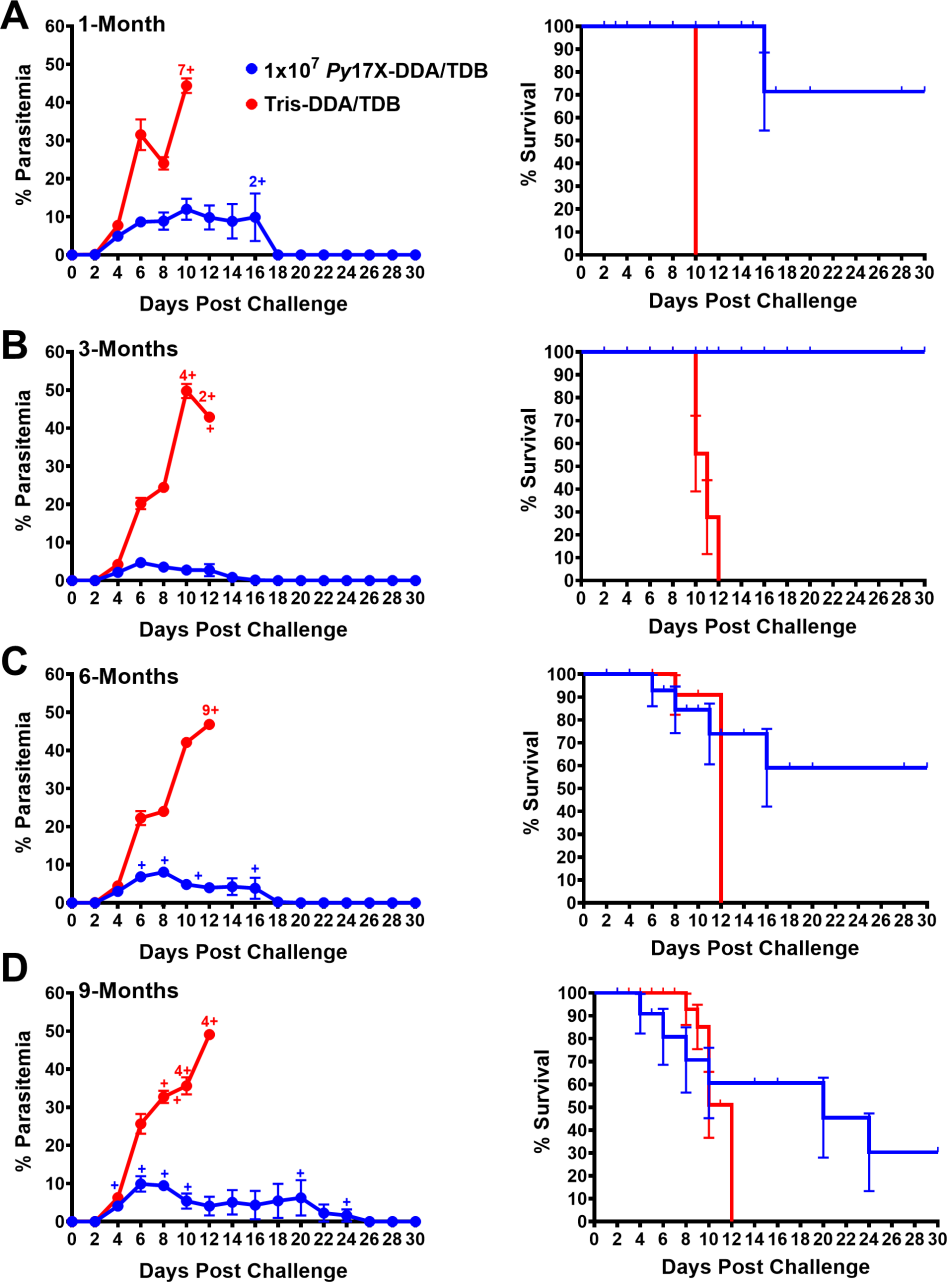
Longevity of protection following homologous challenge in *Py*17X-DDA/TDB vaccinated mice at 1-, 3-, 6-, and 9-month timepoints. Groups of female BALB/c mice (*n* = 10/group) were immunized with 10^7^ *Py*17X-DDA/TDB or Tris-DDA/TDB. Mice were rested for (**A**) 1, (**B**) 3, (**C**) 6, or (**D**) 9 months following the final vaccine dose prior to receiving homologous blood-stage challenge infection with 10^5^ *Py*17X pRBCs i.v. Parasitemia and Kaplan-Meier survival curve analysis plotted as a percent of survival, error bars show standard error of survival probability at each timepoint, were recorded up to 30 days following challenge. All data are expressed as means ± SEM. Cross sign represents euthanized mice.

We then asked whether vaccination would induce enduring protection and challenged vaccinated and control (Tris-DDA/TDB) mice (10 per group) at 1, 3, 6, and 9 months post last vaccine dose. Mice immunized with 10^7^ *Py*17X-DDA/TDB had mean peak parasitemias of 12.0%, 4.71%, 8.05%, and 9.90% at 1, 3, 6, and 9 months, respectively (*P* > 0.05 for differences between groups) (Fig. 3). There were 71%, 100%, 60%, and 30% survival in mice vaccinated with *Py*17X-DDA/TDB and challenged at 1, 3, 6, and 9 months, respectively. All mice vaccinated with the Tris-DDA/TDB developed high parasitemia and succumbed to the infection. These data demonstrate that a *Py*17X vaccine formulated with DDA/TDB provides protection that can last for at least 6 months post-vaccination.

**Fig 4:**
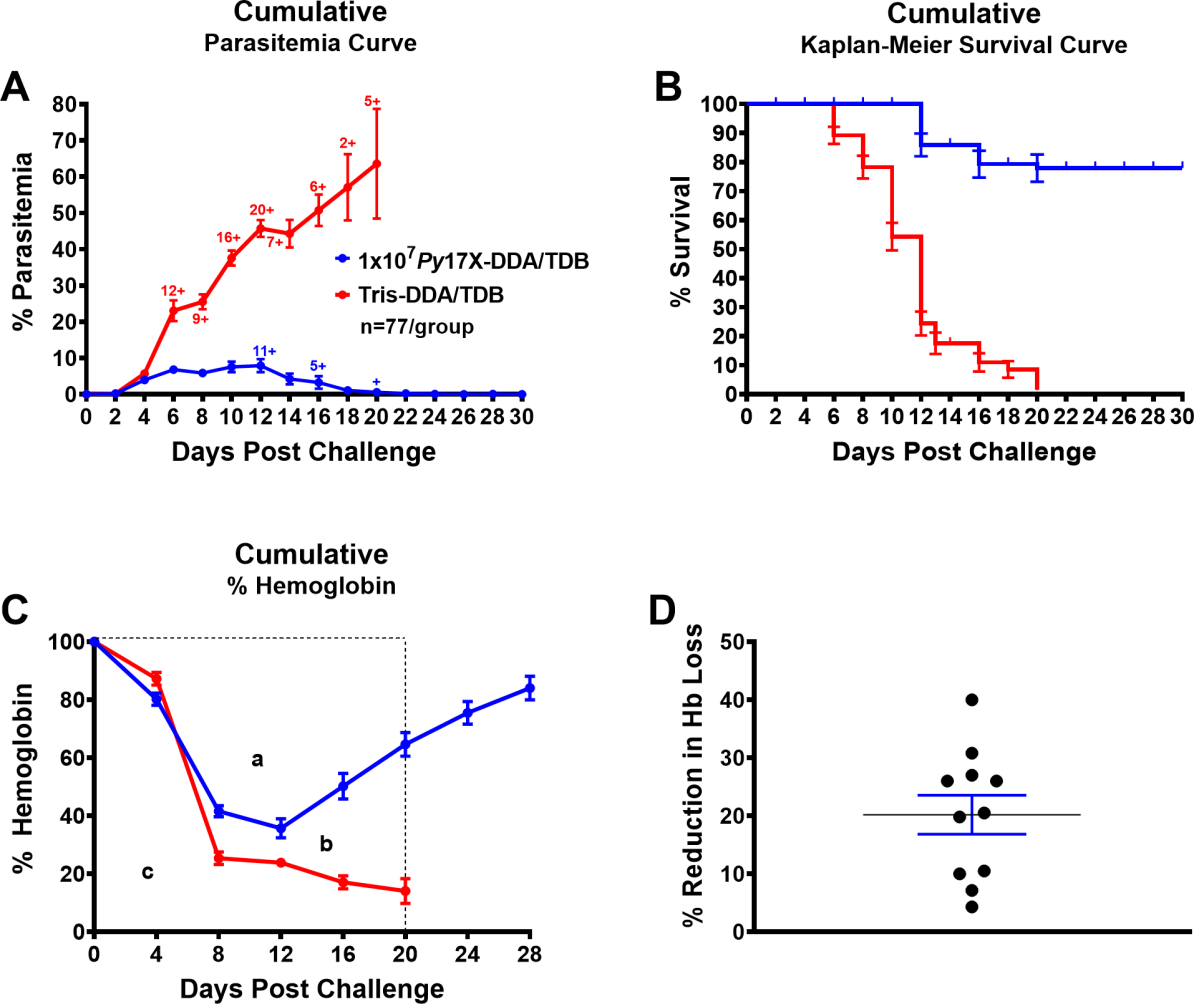
Summary of the protective efficacy of *Py*17X-DDA/TDB vaccine against homologous challenge in female BALB/c mice in 11 different studies. Groups of female BALB/c mice (*n* = 77/group) were immunized with three doses of 10^7^ *Py*17X-DDA/TDB vaccine or Tris-DDA/TDB administered s.c., with each dose 2 weeks apart. Mice were then rested for 4 weeks prior to receiving homologous challenge with 10^5^ *Py*17X pRBCs i.v. Mice were monitored for 30 days following challenge by monitoring (**A**) cumulative parasitemia, (**B**) cumulative Kaplan-Meier survival curve analysis for all mice plotted as a percent of survival, error bars show standard error of survival probability at each timepoint, (**C**) percentage cumulative hemoglobin plotted as percentage of starting Hb, and (**D**) cumulative mean percentage reduction in hemoglobin loss as a result of *Py*17X-DDA/TDB vaccination determined by area under the curve analysis definite integral of polynomial function order 2: [(*b/a + b*)×100)], where *a* = AUC Hb lost in vaccine group, *c* = total AUC in control group, *b* = total AUC vaccine group minus *c*, from day 0 to day 20 post-challenge when all mice in the control group were euthanized. All data are expressed as means ± SEM. Cross sign represents euthanized mice.

We have conducted 11 separate studies where a total of 77 BALB/c mice were vaccinated with 10^7^ *P. yoelii* 17X-killed pRBCs adjuvanted with DDA/TDB and challenged 1 month after the final vaccine dose. In all experiments, mice were monitored every 2 days for parasitemia and every 4 days to estimate hemoglobin levels. Figure 4 shows the mean parasitemia control of the 11 separate experiments, including control groups which received Tris-DDA/TDB (Fig. 4A), the Kaplan-Meier survival plots of all mice (Fig. 4B), cumulative mean percentage hemoglobin (Fig. 4C), and the mean percentage reduction in hemoglobin loss as a result of vaccination (Fig. 4D). The mean peak parasitemia of *Py*17X-DDA/TDB-vaccinated mice was 7.9% ± 0.90%, the percentage survival at day 30 was ~78%, and the mean percentage reduction in hemoglobin loss (monitored from the time of challenge until the last control mouse succumbed) was ~20%. All vaccinated mice that recovered regained more than 80% of their initial hemoglobin levels within 28 days post challenge. It is also worth noting that all vaccinated mice that succumbed were euthanized due to the fact that they had lost 15% of their body weight, according to ethics guidelines, not because of their clinical scores. All control mice succumbed to high parasitemia or clinical scores which exceeded ethical guidelines (see Materials and Methods).

### Immunity against parasitized reticulocytes following vaccination

*P. yoelii* preferentially infects reticulocytes (36, 37). To investigate if vaccine-induced immunity lowered the parasite burden in these cells, we optimized a biotinylation method to track these cells as they exited the bone marrow (38). BALB/c mice were injected with EZ-Link-Sulfo-NHS-Biotin (as described in Materials and Methods), and blood samples were collected at specified days post-biotin injection for flow cytometry analysis (see Fig. S2). Biotin marks all red blood cells at the time of injection, but red blood cells developing after that time will not be marked. We showed that biotin labeled all red blood cells and that these cells diminished in number linearly over time (see Fig. S2).

**Fig 5:**
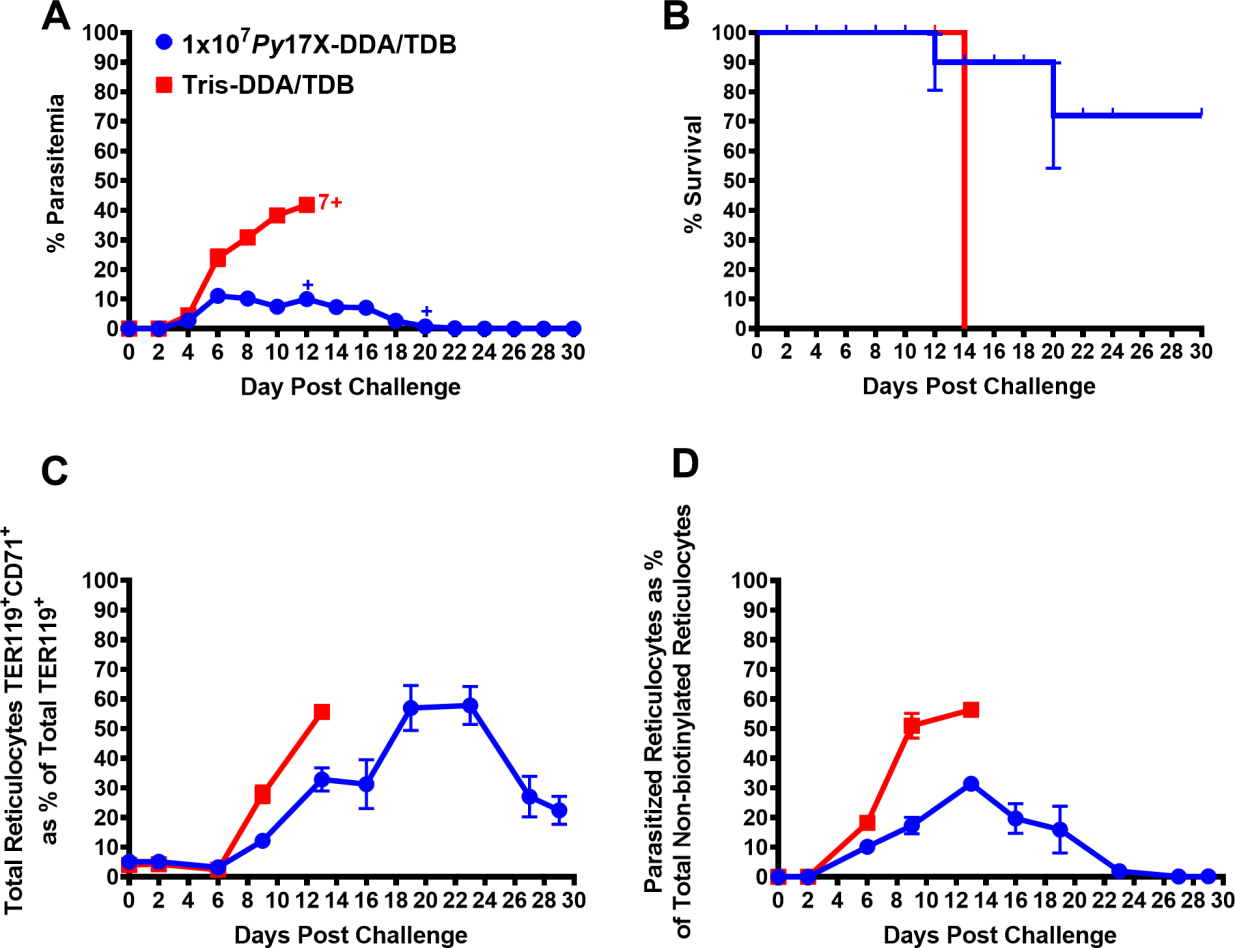
Tracking red blood cells following vaccination and challenge in female BALB/c mice. Groups of BALB/c mice (*n* = 7/group) vaccinated with 10^7^ *Py*17X-DDA/TDB or Tris-DDA/TDB received biotinylating agent i.v. on day −1 relative to challenge day 0. Blood samples were collected at different timepoints days −1, 2, 6, 9, 13, 16, 19, 23, 27, 29 relative to challenge day 0. Samples were stained with streptavidin-PE, TER119 APC (erythroid marker), CD71 FITC (reticulocyte marker), and Hoechst 34580 dye (DNA dye). Monitoring and/or tracking (**A**) parasitemia by microscopy, (**B**) Kaplan-Meier survival curve analysis plotted as a percent of survival, error bars show standard error of survival probability at each timepoint, (**C**) total reticulocytes (TER119^+^CD71^+^) as a percentage of total TER119^+^ population by flow cytometry, and (**D**) parasitized non-biotinylated reticulocytes (TER119^+^CD71^+^PE^-^Hoechst^+^) as a percentage of total non-biotinylated reticulocytes by flow cytometry were recorded/tracked up to 30 days following challenge. All data are expressed as means ± SEM. Cross sign (+) represents euthanized mice.

We then vaccinated mice prior to biotin labeling. Following challenge, parasitemia and survival were similar to what we had previously observed in non-biotinylated mice (Fig. 5A and B). We followed the different cell subsets post challenge infection using streptavidin-PE as a marker of all cells present in the blood at time 0, TER119 (an erythrocyte marker), CD71 (a reticulocyte marker), and Hoechst dye (DNA marker for parasites). We plotted the total number of reticulocytes in the blood in vaccinated and control mice. In both groups, reticulocyte numbers, as a percentage of total red cells, increased from ~2% on day 6 post challenge (Fig. 5C) but increased more rapidly in control mice, likely due to the lower hemoglobin levels in these mice. In vaccinated mice, the reticulocyte numbers peaked at 3 weeks post challenge at ~60% and then rapidly declined. We found that the percentage of new reticulocytes (TER119^+^ CD71^+^ PE^-^) that were infected in vaccinated mice, over the course of the study, was about half the percentage that were infected in control mice (Fig. 5D). Therefore, these data show that vaccine-induced immunity is playing a role in controlling and clearing parasitized reticulocytes following challenge infection in vaccinated mice.

### Immune responses to vaccination and correlates of protection

**Fig 6:**
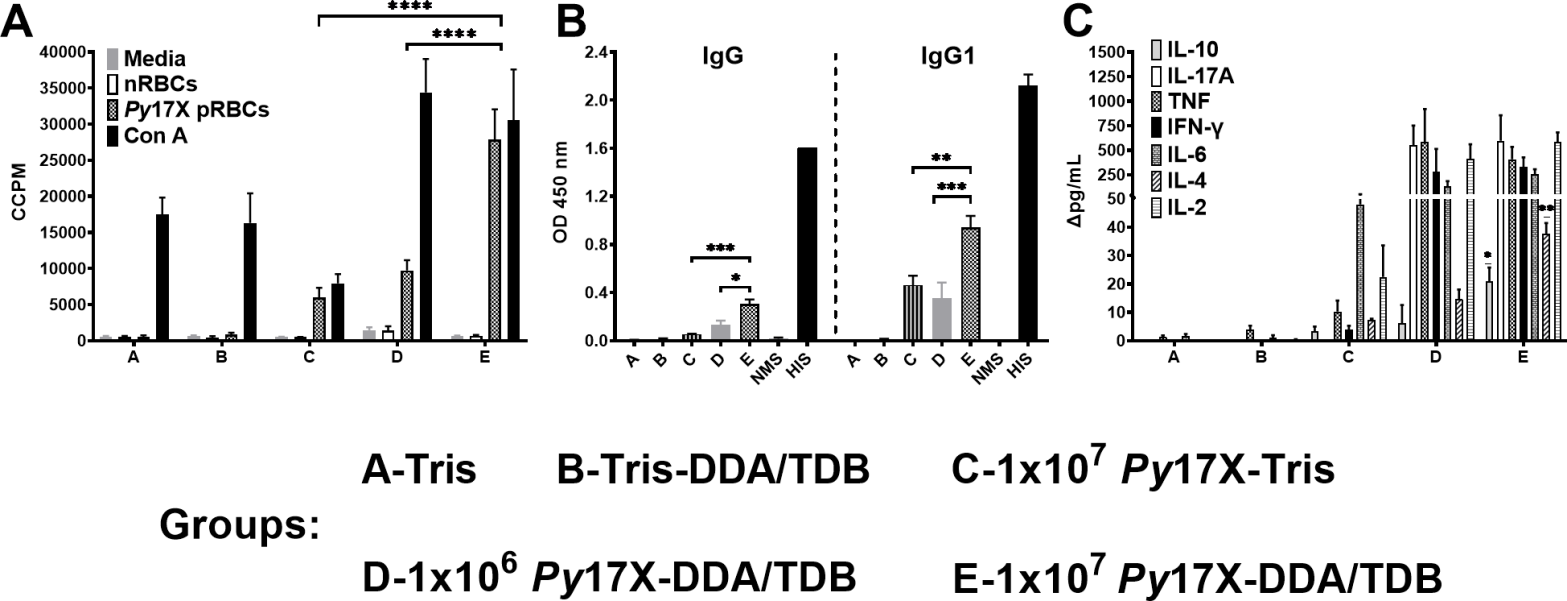
Immunogenicity of a *Py*17X-DDA/TDB whole blood-stage vaccine in inbred mice. Groups of female BALB/c mice (*n* = 10/group) were vaccinated with three doses of Tris, Tris-DDA/TDB, 1 × 10^7^ *Py*17X-Tris, 10^6^ *Py*17X-DDA/TDB, or 10^7^ *Py*17X-DDA/TDB. After 4 weeks of rest, spleens were harvested (*n* = 3/group). Mouse spleen cells were incubated with different stimulants (media, nRBCs, pRBCs, and concanavalin A) in triplicates, and proliferation was estimated through incorporation of [^3^H]thymidine and represented as corrected counts per minute (CCPM). Parasite-specific (**A**) splenocyte proliferative responses, (**B**) IgG, and IgG1 subclass responses in all mice groups were measured by enzyme-linked immunosorbent assay using crude whole *Py*17X parasite antigen, and (**C**) cytokine response assessed using mouse Th1/Th2/Th17 cytokine bead array analysis of the culture supernatants of the splenocyte proliferation assay collected after 54 hours represented as Δpg/mL (nRBCs background subtracted from pRBCs). Pre-challenge sera were tested in duplicate at 1:100 dilution. All data are expressed as means ± SEM. *P < 0.05, **P < 0.01, ***P < 0.001, ****P < 0.0001. NMS, normal mice sera; HIS, hyperimmune mice sera.

Having established that CAF01-adjuvanted vaccination can induce sustained homologous and heterologous protection, we assessed the level and type of immune responses that were induced by vaccination and which were required for protection in inbred mice. BALB/c mice were vaccinated with three doses of 10^6^ or 10^7^ *P. yoelii* 17X -DDA/TDB. Control mice were vaccinated with Tris, Tris-DDA/TDB, or 10^7^ killed *P. yoelii* 17X in Tris, and immune responses assessed 1 month post vaccination. Significant parasite-specific splenic proliferative responses (*P* < 0.0001) (Fig. 6A) and parasite-specific total IgG and IgG1 were detected/measured in vaccinated mice. IgG and IgG1 were significantly higher in mice that received the highest dose of vaccine (*P* < 0.05 and *P* < 0.001, respectively) (Fig. 6B). IgG1 was the only subclass detected. In vaccinated mice, a mixture of Th1/Th2/Th17 cytokines were produced in response to stimulation with homologous pRBCs; however, the responses were similar between mice that received the lower or higher doses of vaccine (Fig. 6C). There were zero to minimal immune responses in mice that received the control vaccine preparations, with the exception of a parasite-specific IL-6 response from spleen cells of mice that received parasite alone in Tris.

**Fig 7:**
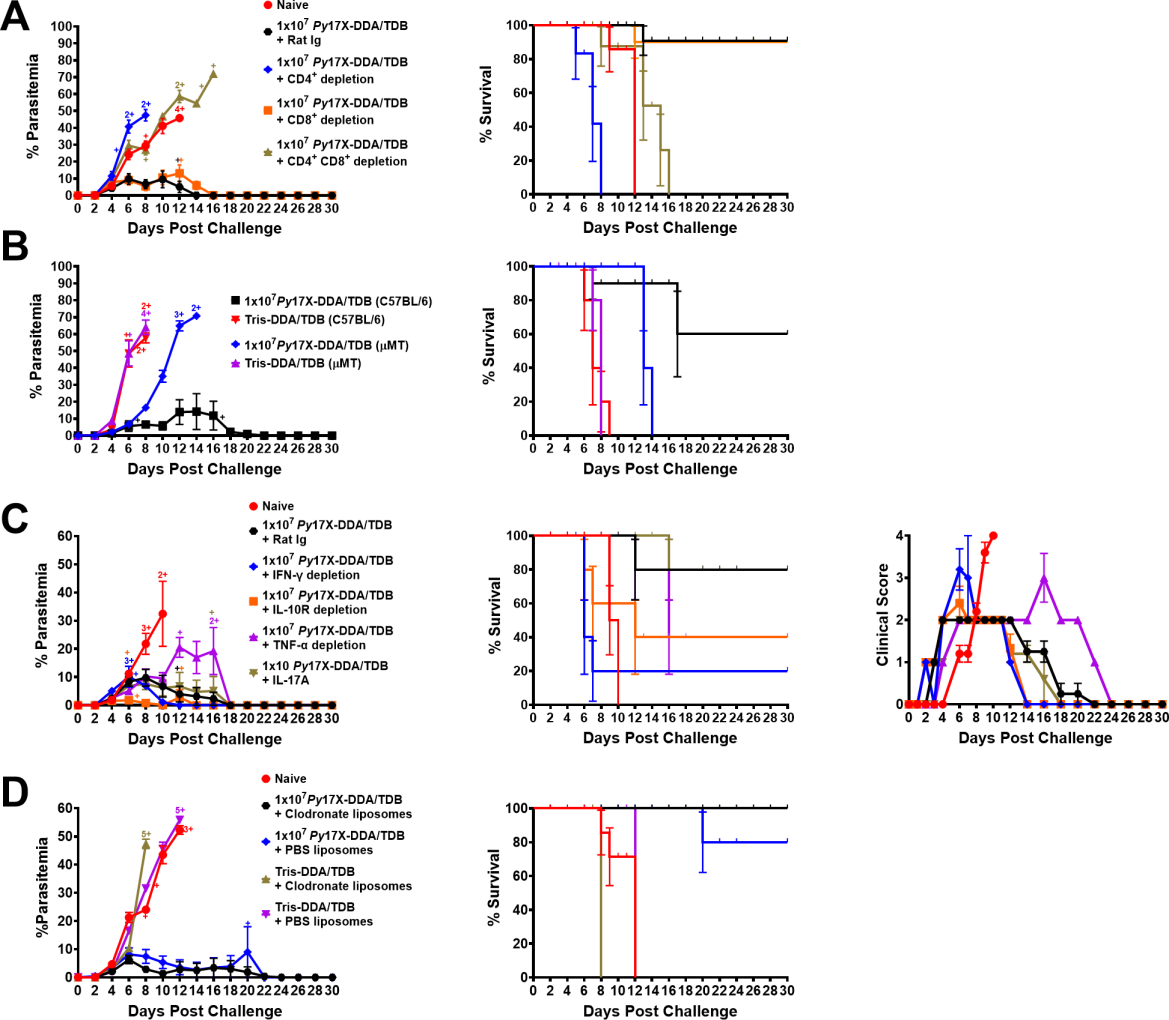
Role of CD4^+^ T-cells, CD8^+^ T-cells, B-cells, cytokines, and macrophages in protective immunity induced by *Py*17X-DDA/TDB vaccine in mice. (**A**) Role of T-cells. Groups of female BALB/c mice (*n* = 5 mice/group) immunized with 10^7^ *Py*17X-DDA/TDB received challenge with 1 × 10^5^ *Py*17X pRBCs 4 weeks after final dose. These mice received injections with rat Ig-, anti-CD4^+^-, or anti-CD8^+^-depleting antibodies on days −2, −1, 4, and 8 relative to challenge on day 0. A positive control group received vaccine and rat Ig. A negative control consisted of naïve-challenged mice only. (**B**) Role of B cells. Groups of female mice (*n* = 5/group) (C57BL/6 or µMT) immunized with 10^7^ *Py*17X-DDA/TDB or Tris-DDA/TDB received challenge with 1 × 10^5^ *Py*17X pRBCs 4 weeks after the final dose. (**C**) Role of cytokines. Groups of female BALB/c mice (*n* = 5/group) were immunized with 10^7^ *Py*17X-DDA/TDB vaccine and received homologous challenge with 10^5^ *Py*17X pRBCs 4 weeks after receiving the final dose. To examine the role of proinflammatory and anti-inflammatory cytokines in protective immunity, mice were injected with 1 mg of rat Ig, anti-interferon gamma (IFN-γ), anti-IL-10R, anti-tumor necrosis factor, or anti-interleukin-17 receptor (IL-17A) antibodies given i.p. at 200 µL on days −1, 1, 4, 7, and 11 relative to challenge on day 0. A positive control group received vaccine and rat Ig. A negative control group consisted of naïve mice that received challenge only. (**D**) Role of macrophages. Groups of female BALB/c mice (*n* = 5 mice/group) were immunized with three doses of 10^7^ *Py*17X-DDA/TDB and received homologous challenge 4 weeks after the final dose with 1 × 10^5^ *Py*17X pRBCs. Vaccinated mice received 200 µL of liposome suspension containing clodronate (5 mg/mL) or PBS i.v. on days −1 and 7 relative to day 0 (challenge). Macrophage depletion was assessed by flow cytometry on days +1 and +9 relative to challenge on day 0. Parasitemia, survival, and clinical scores (cytokine study) were recorded up to 30 days following challenge. All data are expressed as means ± SEM. Cross sign represents euthanized mice.

We examined the critical role of T-cells by performing immuno-depletion studies. Vaccinated BALB/c mice received injections of monoclonal anti-CD4, anti-CD8, both anti-CD4 and anti-CD8 antibodies, or rat immunoglobulin (Ig) as a control, on days −2, −1, 4, and 8 relative to the day of homologous challenge. Unvaccinated naïve mice, which were not immunodepleted, were also included as controls. At the time of challenge, >90% depletion of CD4^+^ T-cells, CD8^+^ T-cells, or both T-cell subsets was confirmed by flow cytometry (data not shown). Vaccinated mice that were depleted of CD4^+^ T-cells alone or depleted of both CD4^+^ and CD8^+^ T-cells developed high parasitemias and succumbed to the infection, similar to the naïve control group (Fig. 7A). Only vaccinated mice that received CD8^+^ T-cell-depleting antibodies alone or rat Ig survived infection with low mean peak parasitemias. These data support the role of CD4^+^ T-cells in protective immunity. To further confirm the role of T-cells, we performed adoptive transfer studies of purified splenic CD4^+^ or CD8^+^ T-cells into immunodeficient severe combined immunodeficiency (SCID) mice. Following challenge, purified CD4^+^ T-cells (1 × 10^6^ or 1 × 10^7^) derived from vaccinated mice were essential for sustained survival of the recipient SCID mice and control of parasitemia compared with mice that received CD4^+^ T-cells from mice vaccinated with Tris-DDA/TDB only (see Fig. S3). Ultimately, however, these CD4^+^ T-cells could not eliminate the parasite, and surviving mice were euthanized 40 days following challenge due to the >15% weight loss clinical criterion. All SCID mice that received purified CD8^+^ T-cells (half-spleen equivalent) from groups of the same donor mice succumbed to infection by day 12 following challenge (see Fig. S4A). These data further confirm the critical role of CD4^+^ T-cells in vaccine-induced immunity.

We next examined the role of B-cells using immunodeficient µMT mice, which lack functional mature B cells (39) and their immunocompetent C57Bl/6 counterparts. Following homologous challenge infection, all vaccinated µMT mice developed high parasitemia (Fig. 7B) similar to the vaccine control groups, albeit the onset of parasitemia was delayed compared with the control groups. All µMT mice were succumbed to infection (Fig. 7B). These data suggested that T-cells alone were controlling infection in the first 6 days post challenge but that B-cells and/or antibody played an important role after that period. However, the adoptive transfer of purified B-cells from *P. yoelii* 17X-DDA/TDB vaccinated mice into immunodeficient SCID mice showed that B-cells alone cannot control the parasite as all recipient mice succumbed to infection 10 days following challenge (see Fig. S4B). Additionally, transfer of purified B-cells into vaccinated µMT mice did not enhance survival or control of parasitemia following homologous challenge infection (data not shown). Passive transfer of immune sera had a minimal effect on controlling parasitemia; however, recipients did survive 4–8 days longer than mice that received naïve serum (see Fig. S5).

Given that CD4^+^ T-cells have a critical role, we undertook further depletion studies to examine the role of key pro-inflammatory and anti-inflammatory cytokines. For these studies, we also monitored clinical scores as described in Materials and Methods, as general signs of inflammation. Vaccinated BALB/c mice received injections of interferon gamma (IFN-γ)-, tumor necrosis factor (TNF)-, or interleukin-17 receptor (IL-17A)-depleting antibodies or IL-10R-blocking antibodies, or rat Ig (control) on days −1, 1, 4, 7, and 11 relative to the day of challenge. Mice that were treated with TNF-depleting antibodies had a higher peak parasitemia (20.5% ± 3.51% vs 9.76% ± 2.98%; *P* = 0.0457, AUC) and ~40% survival compared to mice in the rat Ig control group (~90% survival) (Fig. 7C). Interestingly, vaccinated mice treated with IFN-γ-depleting antibodies had a similar parasitemia curve as the rat Ig control group; however, these mice presented a high clinical score and at an earlier time than the naïve control mice, and only 20% survived (Fig. 7C). Mice treated with IL-10R-blocking antibodies had a significantly lower mean peak parasitemia (2.77% ± 2.77%) than mice in the rat Ig control group and all other groups; however, they had high clinical scores on day 6 post-challenge infection, and 60% succumbed to the infection (Fig. 7C). Mice treated with anti-IL-17A-depleting antibodies had similar parasitemias, clinical scores, and survival as the rat Ig control group (Fig. 7C). Therefore, these data demonstrate that, for this vaccine, pro-inflammatory cytokines (IFN-γ and TNF) are playing important role in controlling parasitemia and promoting survival and that the anti-inflammatory cytokine, IL-10, is a key factor influencing the control of vaccine-induced inflammatory responses.

The inflammatory cytokines, IFN-γ and TNF, can activate other immune cells, such as macrophages, that can directly kill parasites through phagocytosis. We performed depletion studies to determine the role of macrophages in vaccine-induced immunity. BALB/c mice vaccinated with *Py*17X-DDA/TDB or Tris-DDA/TDB were injected with liposomes containing clodronate or phosphate-buffered saline (PBS) on days −1 and 7 relative to challenge day 0. Depletion was confirmed by flow cytometry on days +1 and 9 relative to challenge on day 0 (see Fig. S6). Clodronate is toxic to macrophages, and following ingestion of these liposomes by macrophages via endocytosis, it initiates programmed cell death (apoptosis) (40). Intravenous administration of clodronate liposomes leads to maximum depletion of liver and splenic macrophages in 24 hours (41, 42). Depletion of splenic red pulp, marginal zone, and marginal metallophilic macrophage subsets was confirmed by immunohistochemistry (IHC; see Fig. S7). We observed that *Py*17X-DDA/TDB-vaccinated mice that received clodronate or PBS liposomes both developed low parasitemias, which were not significantly different between groups (*P* > 0.05) and survived challenge (Fig. 7D). Both groups vaccinated with Tris-DDA/TDB that received clodronate or PBS liposomes developed high parasitemias and succumbed to the challenge infection.

### The impact of infection on priming and boosting vaccine-induced immunity

**Fig 8:**
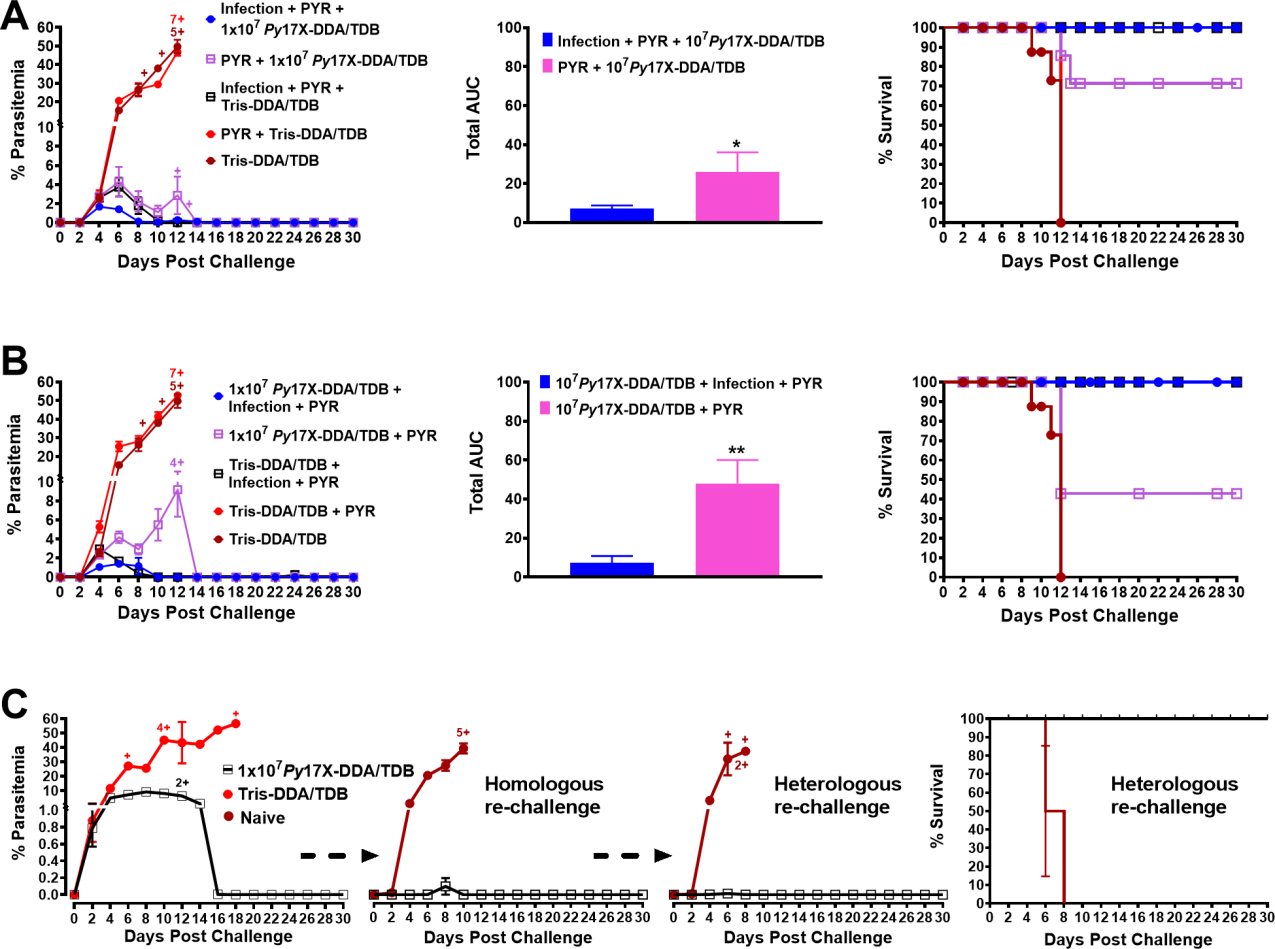
Priming or boosting *Py*17X-DDA/TDB vaccinations with infection drug-cure (IDC) regimen. Female BALB/c mice (*n* = 7/group) immunized with *Py*17X-DDA/TDB vaccine or Tris-DDA/TDB were primed or boosted with i.v. infection (3 × 10^7^ *Py*17X pRBCs) followed by treatment with fast-acting antimalarial drug pyrimethamine (0.2 mg/mouse/day, i.p.) 8 hours post-infection administered for four consecutive days. All mice groups were rested for 2 weeks between IDC and first vaccine dose, 2 weeks between each vaccine doses, 2 weeks between IDC and last vaccine dose, and for 4 weeks between last IDC and challenge infection with 1 × 10^5^ *Py*17X pRBCs. (**A**) Priming and (**B**) boosting *Py*17X-CAF01 vaccinations: parasitemia, parasitemia total area under the curve, and survival following challenge. (**C**) Protective efficacy following homologous and cross-strain rechallenge in *Py*17X/CAF01-vaccinated BALB/c mice. Groups of female BALB/c mice (*n* = 7/group) immunized with 10^7^ *Py*17X-CAF01 received first homologous challenge with 1 × 10^5^ *Py*17X pRBCs 4 weeks after the final vaccine dose, surviving and naïve mice received homologous re-challenge with 1 × 10^5^ *Py*17X pRBCs i.v. on day 71 (*n* = 5/group) and cross-strain re-challenge with 1 × 10^5^ *Plasmodium yoelii* YM pRBCs on day 140 (*n* = 5/group) relative to day 0 initial homologous *Py*17X challenge (*n* = 7/group). Naïve mice were used as controls in both re-challenge studies. Parasitemia following homologous, homologous re-challenge, heterologous re-challenge, and survival following heterologous re-challenge were recorded up to 30 days. All data are expressed as means ± SEM. Cross sign represents euthanized mice. AUC data were analyzed using unpaired *t* test analysis. *P < 0.05, **P < 0.01.

Because vaccines will be mostly administered to individuals who have been previously exposed to malaria, we asked whether the vaccine would be more effective in mice which were previously infected. However, infection of mice is known to induce a strong immunity making it difficult to see an improved response as a result of vaccination. We, thus, gave mice a pulsed infection which was halted with pyrimethamine (PYR) after only 8 hours. Mice never became patently infected during that time. The goal was to give them partial immunity as a result of infection. Two weeks later, some of the mice were vaccinated with the first of three doses of 10^7^ *P. yoelii* 17X-DDA/TDB, whereas other mice that received the pulsed infection were administered Tris-DDA/TDB. All mice received PYR so that the effect of any residual drug would not confound the interpretation of the data. However, there did not appear to be any residual drug effect as control mice which received Tris-DDA/TDB only had near-identical parasitemia curves to control mice that received Tris-DDA/TDB plus PYR (Fig. 8A). Following challenge, vaccinated mice which had previously experienced a pulsed infection showed significantly reduced parasitemia as determined by peak parasitemia and AUC analysis (*P* = 0.0407), and enhanced survival (Fig. 8A) compared to control vaccinated mice that only received PYR. We also asked whether a pulsed infection after *Py*17X-DDA/TDB vaccination would also further boost immunity and similarly found that it did (AUC, *P* = 0.0071) (Fig. 8B).

We thus determined that priming or boosting vaccinations with pulsed infections significantly enhances the protective efficacy of the vaccine against homologous challenge. We showed above (Fig. 2C) that the vaccine could confer protection against a heterologous strain of *P. yoelii*. Given that an infection can boost immunity, we then tested immunity following a homologous re-challenge and a subsequent heterologous challenge (Fig. 8C). Four weeks following vaccination with 10^7^ *Py*17X-DDA/TDB, BALB/c mice were challenged with *P. yoelii* 17XL (Fig. 2B). Mice were protected (~9.2% ± 1.7% mean peak parasitemia; ~70% survival). Following homologous re-challenge ~10 weeks later, the degree of protection had significantly improved with all mice surviving with a peak parasitemia of 0%–0.1%. Following a heterologous re-challenge after a further 20 weeks, the peak parasitemia was <0.1%, and mice were totally protected (100% survival) (Fig. 8C). It is likely that the first infection alone contributed significantly to the level of protection observed against the second (homologous) and third (heterologous) infections; nevertheless, the data show that in a model that mimics a real-world situation, vaccination with a CAF01-adjuvanted killed parasite gives significant protection against a first infection and that this enhances immunity to subsequent infections over a prolonged time frame.

## DISCUSSION

Here, we have demonstrated that a CAF01-adjuvanted whole-parasite blood-stage malaria vaccine induces a strain-transcending protective immunity in rodent models of malaria. The *P. yoelii* 17X-DDA/TDB liposomal vaccine formulation induced protective immunity against homologous and cross-strain heterologous challenge. It was effective in reducing parasite burden in both normocytes and reticulocytes. Vaccine-induced immunity was significantly enhanced by subsequent exposure to malaria; likewise, immunity induced by prior exposure to malaria was enhanced by the vaccine. The vaccine induced both cell-mediated and parasite-specific antibody responses. However, the only detected subclass in the immune sera was IgG1.

While we previously demonstrated that a different liposomal preparation that included a TLR4 agonist (PHAD) and mannose could induce protective immunity (16), that platform has not been developed at good manufacturing practice (GMP) level, and vaccine manufacture requires co-assembly of the encapsulated parasite antigen in the liposome. In contrast, CAF01 is a stand-alone adjuvant formulation (available at GMP), which is mixed with antigen at the point of delivery. It is a clinically tested and safe vaccine adjuvant that has been shown to induce potent humoral and cellular immune responses including priming strong T-cell immunity against intracellular pathogens including *Mycobacterium tuberculosis* (32) and *Chlamydia trachomatis* (34). It has also been tested in subunit vaccines against *P. falciparum* (33, 43). The adjuvant has been shown to trigger potent cell-mediated CD4^+^ T-cell responses (29, 44–46). The immunostimulant (TDB) component of CAF01 is the potentiator of Th1/Th17-biased immune responses (29). In pre-clinical studies, CAF01 has been shown to elicit strong IFN-γ and IL-17 cytokine responses as well as long-lived memory responses (32, 47–49). CAF01 induces IL-17 production through activation of the Syk-FcRγ-Card9-Bcl-10-Malt1 signaling pathway, resulting in the generation of pro-inflammatory cytokines such as IFN-γ, TNF-α, IL-1β, IL-6, and TGF-β (50). This signaling pathway is activated by the interaction between TDB and the C-type lectin receptor MINCLE (51, 52). In keeping with these published findings, we found that the *P. yoelii* 17X-DDA/TDB vaccine induced a mixed Th1/Th2/Th17 cytokine profile as we observed elevated levels of IL-2, IFN-γ, TNF-α, IL-6, and IL-17 cytokines in stimulated spleen cell supernatants post vaccination.

We demonstrated that the vaccine does not protect µMT mice, suggesting B-cells play a role in vaccine-mediated immunity. However, in the first 6 days post challenge, vaccinated µMT mice could control parasite burden as effectively as vaccinated normal mice showing that B-cells are not playing a role in this early stage of infection. However, purified B-cells from immune mice could not transfer protective immunity to immunodeficient SCID mice, demonstrating that vaccine-induced B-cells alone cannot protect. In contrast, adoptively transferred T-cells (into SCID mice) led to prolonged survival, although recipients ultimately succumbed. T-cell immuno-depletion studies demonstrated the critical role of CD4^+^ T-cells. CD8^+^ T-cell depletion did not ablate immunity. The data are consistent with a model in which immunity post challenge is initially and solely mediated by CD4^+^ T-cells. These cells continue to play a role throughout the infection but rely to a degree on antibody to ultimately clear the infection. The data are reminiscent of early studies in *P. chabaudi* that showed that Th-1 cells were initially important in parasite control, but a switch to Th-2 cells was required for parasite clearance (53). We found that Th-1 and Th-2 cytokines were critical for parasite control and survival. IL-10 was shown to play a balancing role. Blocking IL-10 binding to IL-10R led to significantly controlled parasitemia but at a price involving inflammatory side effects in the mice and poor survival.

To further examine the mechanisms of protection, we assessed the roles of macrophages. They were depleted using clodronate liposomes (40). The data indicated, surprisingly, that vaccine-induced immunity is not dependent on splenic macrophages alone; perhaps, certain macrophage subsets and/or populations not depleted by clodronate may play a significant role in protective immunity. It is also possible that other accessory cell types not examined here are playing a critical role. However, further studies will be needed to elucidate the role of various macrophage sub-populations in vaccine-induced immunity.

Studies on cationic adjuvant formulations have shown that vaccine formulations injected subcutaneously or intramuscularly result in the formation of a depot at the site of injection (54) which may be critical to the adjuvant effect. We found that both s.c. and i.m. routes of vaccine administration resulted in similar protective efficacy following challenge infection in BALB/c mice, although there were more elevated levels of IFN-γ produced by spleen cells from mice that received i.m. immunizations.

In summary, this is the first time that a CAF01-adjuvanted asexual whole blood-stage parasite vaccine has been assessed for efficacy and immunogenicity in rodent models of malaria. We report that the vaccine induces a CD4^+^ T-cell-dependent strain-transcending protective immunity in mice against challenge infection, which can be enhanced by subsequent exposure to malaria, thus holding promise for clinical development. The results will inform the transition of this vaccine candidate and adjuvant system into clinical trials for a *P. falciparum* vaccine.

## MATERIALS AND METHODS

### Rodent malaria parasites

*Plasmodium yoelii* 17X and *Plasmodium yoelii* YM were originally obtained from Richard Carter (Edinburgh, UK).

### Preparation of the CAF01 vaccine

To produce parasites for the vaccine formulations, naïve BALB/c, C57BL/6, or Swiss mice were infected with *P. yoelii* 17X pRBCs administered intravenously. Blood was collected by cardiac puncture when parasitemia reached approximately 40% and washed in sterile 1X PBS. The pRBCs were stored without cryopreservation medium at −80°C until ready for use. For preparation of the vaccine, these frozen pRBCs were resuspended in 10 mM TRIS buffer pH 7.4 and subjected to six freeze-thawing cycles using dry ice and a 37°C water bath to render the parasites non-viable and lyse RBCs. It was then stored at −20°C until formulation with DDA/TDB liposomes. For most studies, DDA/TDB liposomes were prepared in-house using published methodology for CAF01 (29). DDA/TDB liposomes formulated with PHAD (Avanti Polar Lipids) were similarly prepared with the PHAD component (at 25 µg/dose/mouse) dissolved in 0.3 mL of chloroform (16). This was then added to the same flask as used for preparing the DDA/TDB liposomes. For the study comparing s.c. and i.m. routes of administration, we used CAF01 provided by SSI (Denmark). For s.c. injections, CAF01 was provided at 2,500 µg DDA/500 µg TDB/mL, and for i.m. injections, CAF01 was provided at 5,000 µg DDA/1,000 µg TDB/mL. Half the volume was used for the i.m. injections. To formulate the pRBC material with the CAF01, *Py*17X-CAF01 or Tris-CAF01 mixtures were vortexed at high speed for 15 s and left at room temperature for 30 min with regular vortexing every 10 min. Following this, the vaccine was administered to mice within 2 hours.

### Immunization and challenge of mice

Mice received vaccine formulations in a volume of 200 µL (s.c.), 100 µL (i.m.), or 30 µL (i.n.) per dose per mouse. The vaccination regimen consisted of three doses administered 2 weeks apart (days 0, 14, and 28, respectively). The vaccination regimen for a study examining the impact of infection drug-cure (IDC) on *Py*17X-DDA/TDB vaccination consisted of one infection with 3 × 10^7^ *Py*17X pRBCs i.v. in a 200 µL volume early morning followed by treatment with the fast-acting antimalarial drug pyrimethamine (Sigma) after 8 hours (0.2 mg/dose/mouse) for four consecutive days. Four weeks after the final vaccine dose or IDC, mice were challenged with 1 × 10^5^ viable homologous or heterologous pRBCs i.v. and were monitored for 30 days.

Challenge inoculum: mice were challenged with 1 × 10^5^ pRBCs in 1× PBS i.v. Following challenge, mice were monitored every 2 days by Giemsa-stained thin blood films and every 4 days by measuring weights and hemoglobin (Hemocue201^+^ Analyzer). Clinical scores were measured daily according to the approved clinical scorecard. Mice that showed signs of severe distress, according to the clinical scoresheet or those that experienced >15% weight loss from the time of challenge, were euthanized using CO_2_ gas or by cervical dislocation. All experiments were blinded from the time of challenge except for immuno-depletion experiments that were blinded following administration of the final dose of the immunodepleting agent.

### Splenocyte proliferation assay

Mouse spleens were harvested aseptically into complete Roswell Park Memorial Institute medium (RPMI 1640 supplemented with 1% L-glutamine, 10% heat-inactivated newborn calf serum, 0.1% gentamicin, and 0.1% 2-mercaptoethanol), manually teased through a 70-µm cell strainer using a sterile syringe plunger, and washed in complete media by centrifuging at 400 g for 5 min at 4°C. The RBCs were then lysed twice using 5 mL Gey’s lysis buffer. Splenocytes (4 × 10^5^ cells/well) were then seeded into 96-well U-bottomed plates in a volume of 100 µL and cultured in the presence of concanavalin A (10 µg/mL) (positive control) (Sigma Aldrich), complete media (negative control), normal mouse RBCs (5 × 10^5^ nRBCs/100 µL/well) or viable pRBCs (5 × 10^5^ pRBCs/100 µL/well) at 37°C for 72 hours. A portion of the culture supernatants were removed in the last 18 hours of culture and frozen at −80°C for soluble cytokine analysis at this time, 1 µCi of [^3^H]thymidine was added per well to assess splenocyte proliferation (Perkin Elmer). Following the 72-hour culture period, the plates were frozen at −80°C. Later, the thawed plates were harvested onto MicroBeta glass fiber filter mats (Wallac, USA). After air drying the filter mats, [^3^H]thymidine incorporation was measured using a Microbeta2 Microplate Counter (Perkin Elmer) to obtain radioactivity counts per minute, expressed as corrected counts per minute.

### Cytokine profiling using cytokine bead arrays

The mouse Th1/Th2/Th17 Cytometric Bead Array Kit (BD Biosciences, Australia) was used to assess soluble cytokine production in pooled culture supernatants from triplicate wells of the splenocyte proliferation assay. The manufacturer’s guidelines were followed with minor adjustments as previously described (15). Samples were acquired using a BD LSR Fortessa flow cytometer (BD Biosciences), FACS DIVA software version 6 (BD Biosciences), and data analysis was performed using FCAP Array software version 3.0.1 (BD Biosciences).

### Detection of parasite-specific antibodies using ELISA

An indirect enzyme-linked immunosorbent assay was used for the detection of parasite-specific antibodies in the sera of vaccinated mice. Flat-bottomed 96-well MaxiSorp immunoplates (Nunc) were coated overnight at 4°C with 10 µg/mL *P*. *yoelii* 17X crude antigen in bicarbonate coating buffer (pH 9.6). Plates were blocked for 90 min at 37°C with 10% blocking buffer (skim milk in PBS/0.05% Tween-20 [Chem Supply]). Plates were washed twice with wash buffer (1× PBS/0.05% Tween-20) and patted dry. Pre-diluted sera from vaccinated mice were then added, and the plates incubated for 90 min at 37°C followed by six washes. Goat anti-mouse IgG-horseradish peroxidase (IgG-HRP) (Invitrogen), IgG1-HRP, IgG2a-HRP, IgG2b-HRP, or IgG3-HRP (all Thermo Fisher Scientific) was diluted in 5% blocking buffer at 1:3,000 dilution and added to plates for a 90-min incubation at 37°C. After six washes, tetramethylbenzidine substrate (OptEIA BD Biosciences) was added to the wells followed by a 10-min incubation in the dark at room temperature. The reaction was stopped by the addition of 1M sulfuric acid, and absorbance was determined at 450 nm using an xMark microplate spectrophotometer (Bio-Rad).

### Passive transfer of sera

Donor BALB/c female mice were either (i) vaccinated with the 1 × 10^7^ *Py*17X-DDA/TDB vaccine, (ii) received infection drug-cure consisting of three cycles of 1 × 10^5^ *Py*17X pRBCs followed by a 4-day treatment regimen with pyrimethamine (Sigma) (0.2 mg/dose/mouse), or (iii) pyrimethamine only. Mice were rested for 4 weeks between each IDC cycle, and drug treatment was initiated when parasitemia reached ~20% parasitemia. Serum collection commenced 2 weeks after the final IDC and was pooled. Naïve BALB/c-recipient mice received 500 µL of the respective sera on days −1, 0, and 1 relative to homologous challenge with 1 × 10^5^ *Py*17X pRBCs on day 0.

### Depletion and blocking studies

Depletion of different cell populations (CD4^+^ and CD8^+^ T-cells), cytokines (interferon gamma, tumor necrosis factor alpha, and IL-17A, IL-10 was inhibited by using IL-10R blocking antibody), and macrophages was carried out following vaccination. All depletion experiments (except for the macrophage study) had a control group that were given equivalent amounts of rat Ig (Sigma Aldrich) as per the administration schedule for each study. Naïve unvaccinated undepleted mice were included in each depletion study.

For T-cell depletions, vaccinated mice received 250 µg of anti-CD4^+^ (clone GK1.5) (Bio X cell) or anti-CD8^+^ (clone 53–5.8) (Bio X cell) antibodies intraperitoneally on days −2, −1, 4, and 8 relative to challenge (day 0). Depletion was confirmed by flow cytometry on days 1, 9, and 16 following homologous challenge. Splenocytes were stained with CD3-V450 (clone 17A2, BD Horizon), CD4-V500 (clone RM4-5, BD Horizon), and CD8-PerCpCy5.5 (clone 53.6.7, BD Pharmingen).

To deplete macrophages, vaccinated mice received 200 µL of clodronate liposome (5 mg/mL) or PBS liposome suspensions (all from Liposoma, Amsterdam, the Netherlands) i.v. on days −1 and 7 relative to challenge on day 0. Depletion was confirmed by flow cytometry on days 1 and 9 relative to challenge. Splenocytes were stained with CD11c-FITC (clone HL3) and F4/80-PE (clone T45-2342) (BD Biosciences) and a Live/Dead stain (Draq7) (Invitrogen). Depletion was further confirmed by IHC on day 10. Aseptically harvested spleen tissues were embedded in optical coherence tomography mold and kept frozen at −80°C. Briefly, 20 µM sections of the spleen were cut on a Leica Cryostat (Leica Biosystems). The sections were air dried overnight at RT and then used or frozen at −20°C. For labeling, sections were allowed to reach RT before fixing in 100% ice cold acetone for 8 min and encircled with hydrophobic pen. Sections were washed twice in PBS and then blocked in blocking buffer (Agilent: X090930-2) for 40 min at RT, thereafter, stained with antibodies (CD169-BV421 [clone 3D6.112] [BioLegend], F4/80-AF594 [clone BM8] [BioLegend], CD11c-AF488 [clone N418] [BioLegend], CD3e-AF660 [clone 17A2] [Invitrogen], all diluted at 1:200) made up in 2% donkey serum and incubated for 45 min at RT. Sections were then washed three times in PBS, mounted in pro-long gold-mounting medium (Invitrogen), and imaged on a Zeiss LSM780 confocal microscope.

For cytokine depletions, vaccinated mice received 1 mg of anti-IL-17A (clone 17F3), anti-IL-10R (CD210, clone 1B1.3a), anti-TNF (clone XT3.11), or anti-IFN-γ antibody (clone XMG1.2) (all Bio-X-cell) intraperitoneally on days −1, 1, 4, 7, and 11 relative to challenge on day 0.

### *In vivo* adoptive transfer studies

The *in vivo* adoptive transfer studies involved isolation, purification, and transfer of CD4^+^ T-cells, CD8^+^ T-cells, or B-cells from immune donor mice into recipient mice. Antibody-labeled magnetic beads were used to purify B-cells and T-cells (from the spleens of BALB/c donor mice using QuadroMACS LS columns [Miltenyi Biotec] and respective purification kits [mouse CD4^+^ T-cell isolation kit, mouse CD8a^+^ T-cell isolation kit, and mouse Pan B-cell isolation kit II] [Miltenyi Biotec]) according to the manufacturer’s instructions. The purity of the cell populations was confirmed by flow cytometry prior to the adoptive transfer. Purified cells were stained with CD3-V450 (Clone 17A2, BD Horizon), CD4-FITC (clone GK1.5, BioLegend), CD8-PECy7 (clone 53-6.7, eBioscience, Invitrogen), and CD19-Alexa 647 (clone 6D5, BioLegend). Data were analyzed using FlowJo software version 10. Purified cells (10^6^ or 10^7^ for CD4^+^ T-cells and B-cell transfer, or half-spleen equivalent CD8^+^ T-cells) were resuspended in sterile 1× PBS and transferred via i.v. injection into recipient immunodeficient (SCID) mice on day −1 prior to challenge on day 0 with 1 × 10^5^ *Py*17X pRBCs. Mice were then monitored for at least 30 days following challenge. Purified B cells (whole spleen equivalent) from naïve C57BL/6 mice were transferred into immunodeficient naïve µMT mice 4 days prior to administering the first vaccine dose of 1 × 10^7^ *Py*17X-DDA/TDB or Tris-DDA/TDB.

### Tracking red blood cells following vaccination

Female BALB/c mice were immunized with 1 × 10^7^ *Py*17X-DDA/TDB vaccine or Tris-DDA/TDB. Mice were injected with EZ-Link Sulfo-NHS-Biotin in sterile 1× DPBS (1 mg/200 µL/mouse as previously reported in reference 38) i.v. on the day prior to challenge (day −1 relative to day 0 post challenge). Naïve unvaccinated and uninfected biotin-treated mice were used as the control group. Blood was collected via tail bleed on days −1, 2, 6, 9, 13, 16, 19, 23, 27, and 30 post challenge. A step-wise protocol was used to stain the blood with streptavidin-PE (BD Pharmingen, BD Biosciences), anti-mouse CD71-FITC (clone R17217) (Invitrogen), TER119-APC (clone TER-119) (BioLegend), and Hoechst 34580 (Sigma). Six microliters of blood were collected via tail bleed and dissolved in 244 µL of sterile 1× DPBS, vortexed briefly, and centrifuged at 1,000 × *g* for 4 min at room temperature. The supernatant was removed, and the blood pellet was stained with streptavidin-PE (BD Pharmingen, BD Biosciences) in a 50 µL staining volume for 5 min at room temperature. After the incubation, samples were washed with 300 µL sterile 1× DPBS using the same centrifugation settings. Next, the cell pellet was stained with anti-mouse CD71-FITC (Invitrogen) -and anti-mouse TER119-APC (BioLegend) in a 50-µL staining volume on ice for 15 min and washed via centrifugation. Lastly, the pellet was stained with the DNA dye Hoechst 34580 (Sigma) in a 50-µL staining volume for 10 min at room temperature. At the end of the final staining, the sample was washed with 400 µL sterile 1× DPBS centrifuged at 1,000 × g for 4 min at room temperature. The pellet was then resuspended in 300 µL of 1× DPBS. For each timepoint, samples were acquired on the LSR Fortessa FACS Diva flow cytometer (BD Biosciences) and analyzed using FACS Diva software version 6 (BD Biosciences) and FlowJo software 10.

### Statistics

Statistical analyses were performed using Graph pad prism software version 9.4.1. All data are expressed as arithmetic mean ± standard error of mean. To compare study groups and controls, data were analyzed using unpaired Mann-Whitney *U* test, unpaired *t* test, or ANOVA unless stated otherwise. A *P*-value of <0.05 was considered significant for all analyses.

## SUPPLEMENTAL MATERIAL

Fig. S1 mbio.02547-23-s0001.tif Immunogenicity and protective efficacy of a Py17X-DDA/TDB vaccine formulated with or without PHAD.

Fig. S2 mbio.02547-23-s0002.tif Tracking RBCs following vaccination and challenge in female BALB/c mice.

Fig. S3 mbio.02547-23-s0003.tif Protective efficacy of adoptively transferred purified immune CD4^+^ T cells into immunodeficient SCID mice.

Fig. S4 mbio.02547-23-s0004.tif Protective efficacy of adoptively transferred purified immune CD8^+^ T cells or B cells into immunodeficient SCID mice.

Fig. S5 mbio.02547-23-s0005.tif Protective efficacy of passively transferred immune sera into naïve female BALB/c mice.

Fig. S6 mbio.02547-23-s0006.tif Gating strategy and confirming macrophage (F4/80^+^/CD11c^+^) depletion by flow cytometry.

Fig. S7 mbio.02547-23-s0007.tif Confirming depletion of red pulp, marginal zone, and marginal metallophilic macrophage subsets by immunohistochemistry.

Supplemental File mbio.02547-23-s0008.docx Supplemental figure legends.

ASM does not own the copyrights to Supplemental Material that may be linked to, or accessed through, an article. The authors have granted ASM a non-exclusive, world-wide license to publish the Supplemental Material files. Please contact the corresponding author directly for reuse.
