# Supplementary figures and images for: A CAF01-adjuvanted whole asexual blood-stage liposomal malaria vaccine induces a CD4+ T-cell-dependent strain-transcending protective immunity in rodent models
Source: mBio. 2023 Nov 14;14(6):e02547-23. doi: 10.1128/mbio.02547-23 (PMC10746282; doi:10.1128/mbio.02547-23)

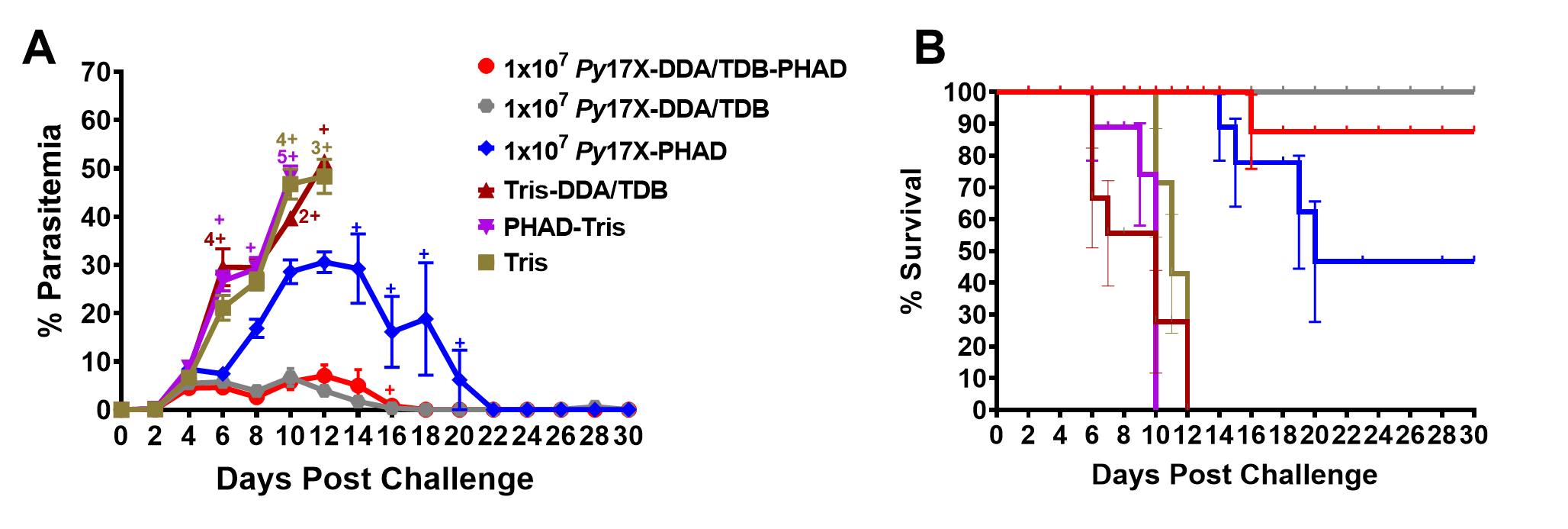

Supplement: Fig. S1 — Immunogenicity and protective efficacy of a Py17X-DDA/TDB vaccine formulated with or without PHAD. [file mbio.02547-23-s0001.tif]

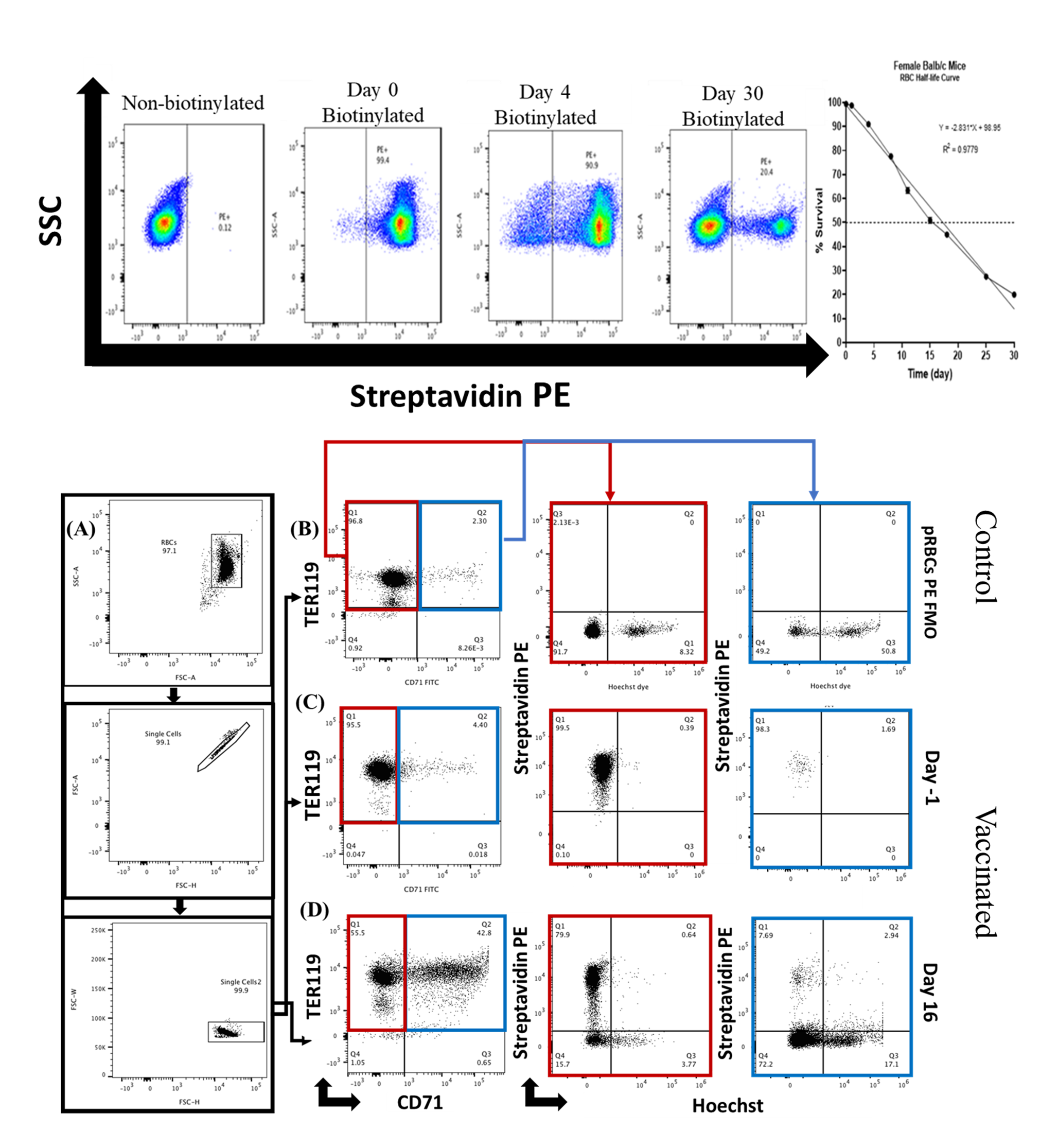

Supplement: Fig. S2 — Tracking RBCs following vaccination and challenge in female BALB/c mice. [file mbio.02547-23-s0002.tif]

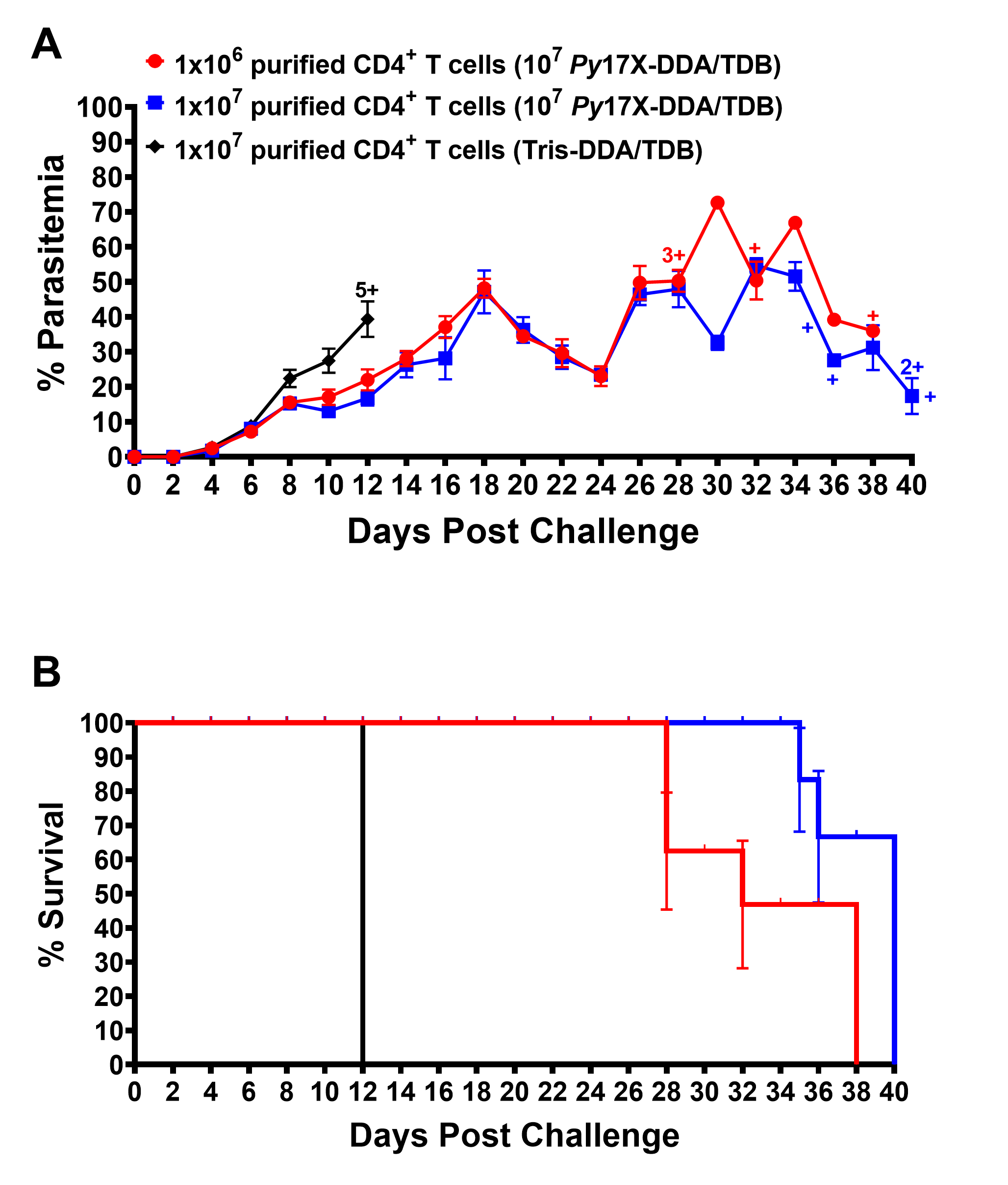

Supplement: Fig. S3 — Protective efficacy of adoptively transferred purified immune CD4+ T cells into immunodeficient SCID mice. [file mbio.02547-23-s0003.tif]

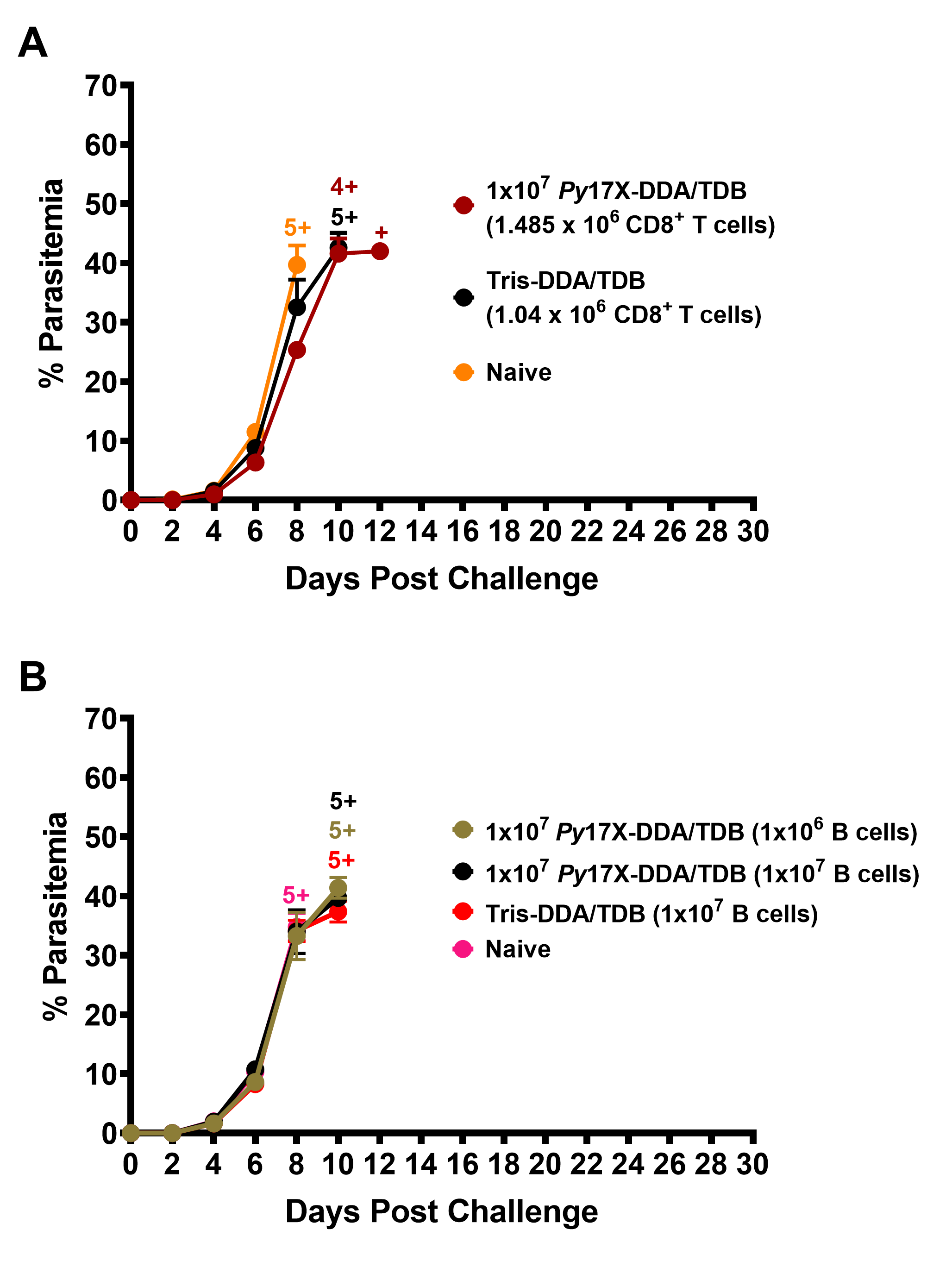

Supplement: Fig. S4 — Protective efficacy of adoptively transferred purified immune CD8+ T cells or B cells into immunodeficient SCID mice. [file mbio.02547-23-s0004.tif]

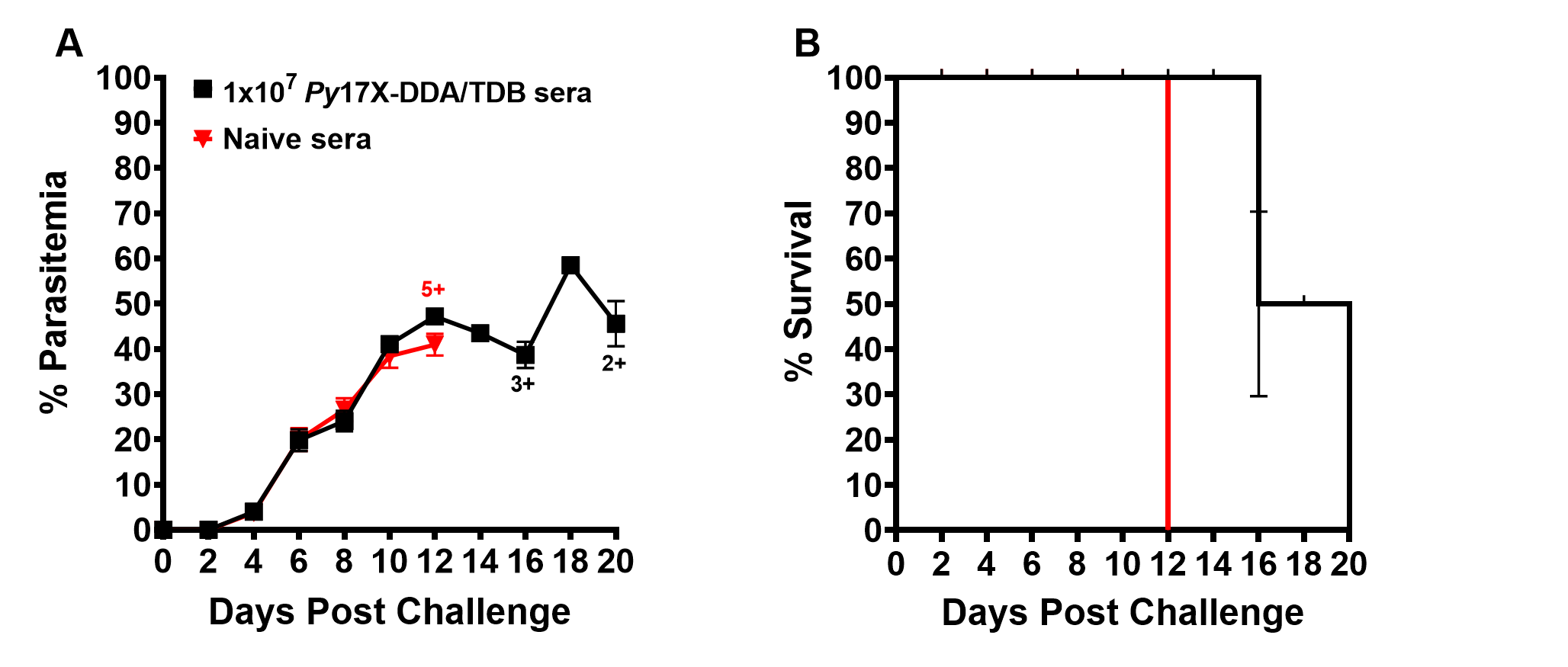

Supplement: Fig. S5 — Protective efficacy of passively transferred immune sera into naïve female BALB/c mice. [file mbio.02547-23-s0005.tif]

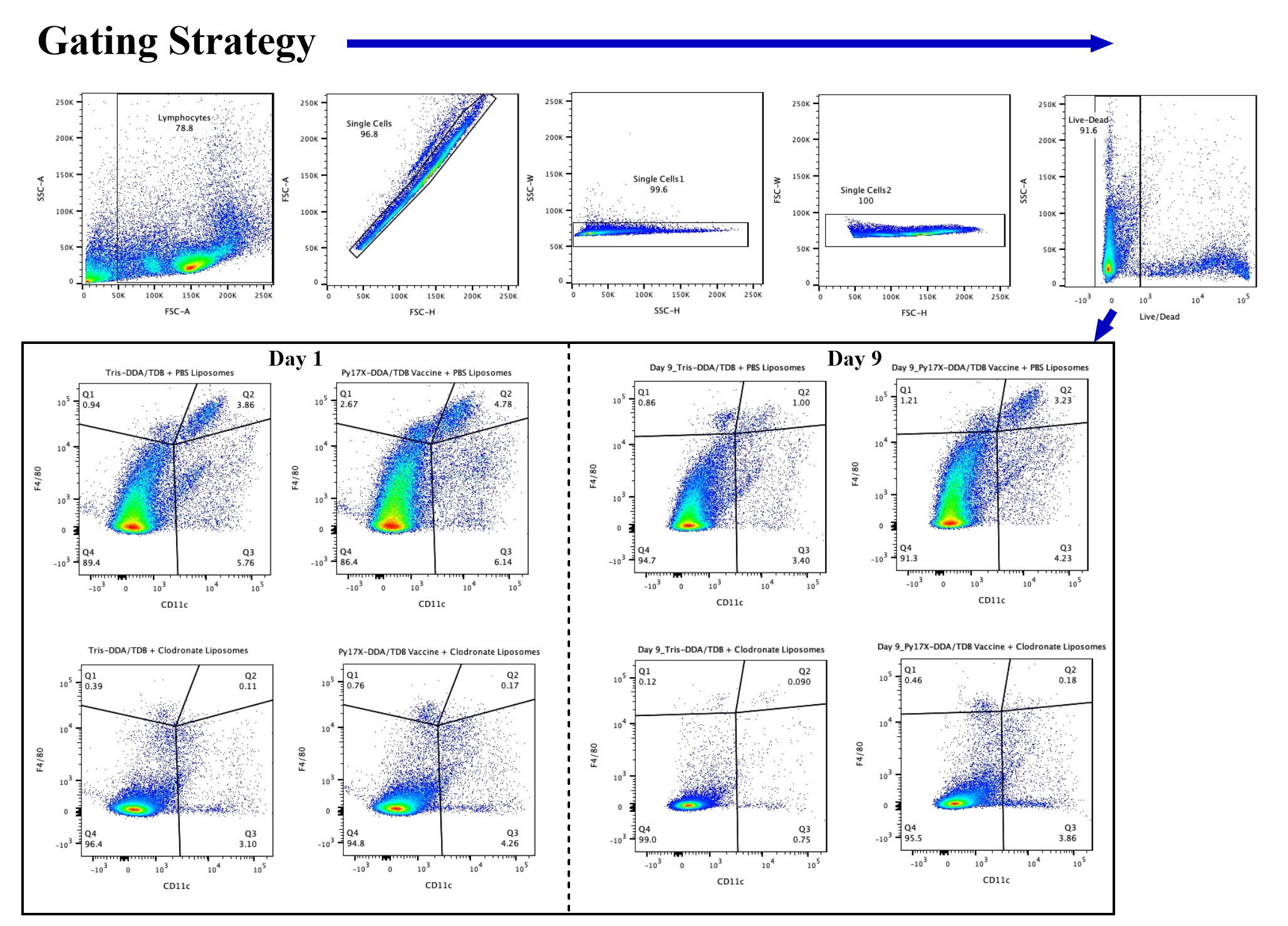

Supplement: Fig. S6 — Gating strategy and confirming macrophage (F4/80+/CD11c+) depletion by flow cytometry. [file mbio.02547-23-s0006.tif]

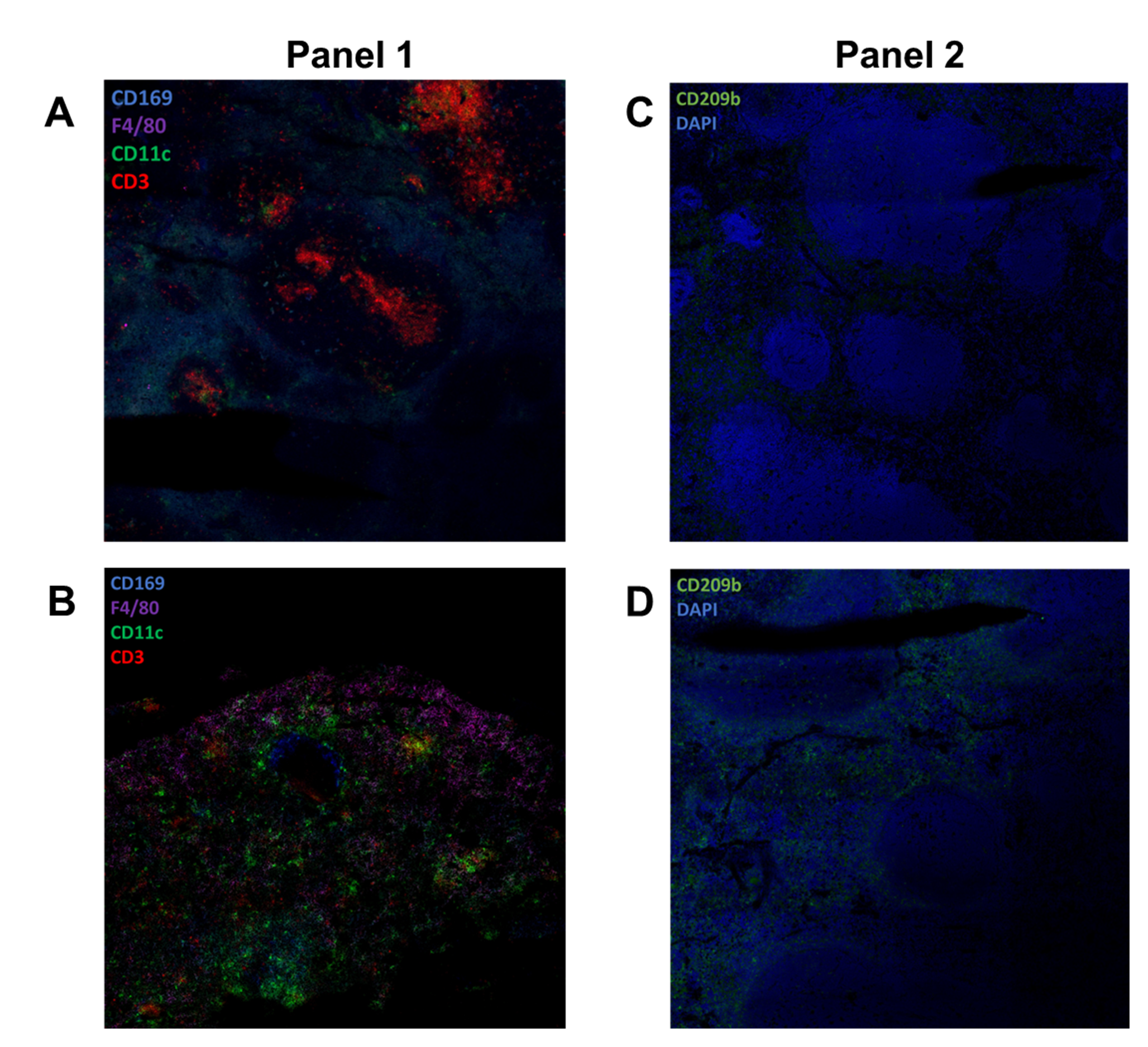

Supplement: Fig. S7 — Confirming depletion of red pulp, marginal zone, and marginal metallophilic macrophage subsets by immunohistochemistry. [file mbio.02547-23-s0007.tif]
