# Supplementary material for: A CAF01-adjuvanted whole asexual blood-stage liposomal malaria vaccine induces a CD4+ T-cell-dependent strain-transcending protective immunity in rodent models
Source: mBio. 2023 Nov 14;14(6):e02547-23. doi: 10.1128/mbio.02547-23 (PMC10746282; doi:10.1128/mbio.02547-23)
Supplement: Supplemental File — Supplemental figure legends. [file mbio.02547-23-s0008.docx]

A CAF^®^01-adjuvanted whole asexual blood-stage liposomal malaria vaccine induces a CD4^+^ T cell-dependent strain-transcending protective immunity in rodent models

Winter A. Okoth,^a^ Mei-Fong Ho,^a^ Mehfuz Zaman,^a^ Emily Cooper,^a^ Priyanka Som,^a^ Mark Burgess,^a^ Maddison Walton,^a^ Reshma J. Nevagi,^a^ Lynette Beattie,^b^ Declan Murphy,^b^ Danielle I. Stanisic,^a*^ Michael F. Good ^a*#^

^a^Institute for Glycomics, Griffith University, Southport, Queensland, Australia.

^b^The Peter Doherty Institute for Infection and Immunity, University of Melbourne, Melbourne, Victoria, Australia

Running Head: CAF^®^01-adjuvanted whole blood-stage malaria vaccine

*These authors contributed equally to the study.

#Address correspondence to: M.F.G [michael.good@griffith.edu.au](mailto:michael.good@griffith.edu.au)

Authorship was determined based on the level of contribution and/or seniority.

**SUPPLEMENTAL FIGURE LEGENDS**

**Figure S1: Immunogenicity and protective efficacy of a *Py*17X-DDA/TDB vaccine formulated with or without PHAD**. Groups of BALB/c mice (n=10 mice/group) were immunized with 10^7^ *Py*17X-DDA/TDB formulated with or without PHAD, or control formulations. Four weeks after the 2^nd^ booster dose, mice were challenged with 1x10^5^ *Py*17X pRBCs i.v. **(A)** parasitemia, and **(B)** Kaplan-Meier survival curve (plotted as percent of survival, error bars show standard error of survival probability at each timepoint) were recorded up to 30 days following challenge. All data are expressed as means ± SEM. Cross sign (+) represents euthanized mice.

**Figure S2: Tracking RBCs following vaccination and challenge in female BALB/c mice.** Groups of naïve mice (n=10) received biotinylating agent i.v. and blood samples collected at different timepoints and stained with Streptavidin PE (biotin marker) were analyzed using flow cytometer. Red blood cell lifespan was determined by linear regression analysis. ***Representative flow cytometry gating strategy used for defining parasitized normocytes and reticulocytes subpopulations in vaccinated and challenged mice*.** Blood samples were collected on days -1, 2, 6, 9, 13, 16, 19, 23, 27, 29 post-challenge infection **(A)** RBCs were distinguished from other cells based on size and structural complexity using the measure of forward scatter (FSC) and side scatter (SSC). To gate on singlets only, doublets were excluded from the analysis using the FSC height versus FSC area gating. **(B)** Biotinylated mouse infected with *Py*17X pRBCs (control). Parasitized RBCs (PE FMO) stained with TER119 APC (erythrocyte marker), CD71 FITC (reticulocyte marker), and Hoechst dye (DNA marker). Gating on TER119^+^CD71^-^ population (red arrow) was used to identify parasitized normocytes/RBCs distinguished as TER119^+^CD71^-^PE^-/+^Hoechst^+^. Gating on TER119^+^CD71^+^ population (blue arrow) was used to identify parasitized reticulocytes distinguished as TER119^+^CD71^+^PE^-/+^Hoechst^+^. **(C & D)** Female BALB/c mouse vaccinated with 3 doses of 10^7^ *Py*17X-CAF01, received biotinylating agent on day -1 relative to challenge day 0 with 1x10^5^ *Py*17X pRBCs. Cells were stained with TER119 (erythroid marker), CD71 FITC (reticulocyte marker), Streptavidin PE (biotin marker), and Hoechst dye (DNA marker). The same gating strategy used for pRBCs PE FMO was applied on day-1 and day 16 timepoints to identify parasitized normocytes and reticulocytes. *FMO = Fluorescence Minus One.

**Figure S3: Protective efficacy of adoptively transferred purified immune CD4^+^ T cells into immunodeficient SCID mice.** Groups of ‘donor’ female BALB/c mice were immunized with three doses of 10^7^ *Py*17X-DDA/TDB or Tris-DDA/TDB each dose given two weeks apart. Spleens were harvested from these mice and CD4^+^ T cells were purified using a CD4^+^ T cell isolation kit (Miltenyi). Purified CD4^+^ T cells (1x10^6^ or 1x10^7^) were adoptively transferred into ‘recipient’ immunodeficient naïve SCID mice on day -1 prior to homologous challenge on day 0 with 1x10^5^ *Py*17X pRBCs. **(A)** Parasitemia and **(B)** Kaplan-Meier survival curve (as a percent of survival, error bars show standard error of survival probability at each timepoint) were recorded up to 40 days following challenge. All data are expressed as mean ± SEM. Cross sign (+) represents euthanized mice. 1x10^6^ vaccine group: mice were euthanized due to clinical scores and >15% weight loss (n=3), due to >15% weight loss (n=1), and clinical scores (n=1). 1x10^7^ vaccine group: mice were euthanized due to clinical scores (n=1), >15% weight loss (n=3), and monitoring period endpoint (n=1).

**Figure S4:** **Protective efficacy of adoptively transferred purified immune CD8^+^ T-cells or B-cells into immunodeficient SCID mice.** Groups of female BALB/c ‘donor mice’ were immunized with 10^7^ *Py*17X-DDA/TDB or Tris-DDA/TDB. Spleens were harvested from these mice and CD8^+^ T cells or B-cells were purified using CD8a^+^ T cell isolation kit or mouse Pan B cell isolation kit II (Miltenyi), respectively. Purified CD8^+^ T cells (~half spleen equivalent/mouse) or B cells (1x10^6^ or 1x10^7^) were adoptively transferred into ‘recipient’ immunodeficient naïve SCID mice on day -1 prior to homologous challenge on day 0 with 1x10^5^ *Py*17X pRBCs. **(A)** Parasitemia in CD8^+^ T-cell recipients and **(B)** parasitemia in B-cell recipient SCID mice were recorded. All data are expressed as mean ± SEM. Cross sign (+) represents euthanized mice. CD8^+^ T cell recipient mice in the vaccine group were euthanized due to clinical scores (n=4), and/or >15% weight loss (n=1). B-cell recipient mice in all groups were euthanized due to clinical scores.

**Figure S5:** **Protective efficacy of passively transferred immune sera into naïve female BALB/c mice**. Naïve mice (n=5/group) received 500 µl of sera from “donor” mice immunized with 3 doses of 10^7^ *Py*17X-DDA/TDB or control naïve sera on days -1, 0, and 1 relative to homologous blood-stage challenge infection with 1x10^5^ *Py*17X pRBCs i.v. **(A)** Parasitemia and **(B)** Kaplan-Meier survival curves (plotted as percent of survival, error bars denote standard error of survival probability at each timepoint) were recorded. All data were expressed as mean ± SEM. Cross sign (+) indicates the number of mice that were euthanized.

**Figure S6: Gating strategy and confirming macrophage (F4/80^+^/CD11c^+^) depletion by flow cytometry.** BALB/c mice were depleted of macrophages on days -1 and +7 relative to challenge on day 0. Spleen cells were stained with F4/80 and CD11c and depletion confirmed by flowcytometry on days ^+^1 and ^+^9 relative to challenge day 0.

**Figure S7: Confirming depletion of red pulp, marginal zone and marginal metallophilic macrophage subsets by immunohistochemistry.** BALB/c mice immunized with 3 doses of 10^7^ *Py*17X-DDA/TDB or Tris-DDA/TDB were depleted of macrophages on days ^-^1 and ^+^7 relative to challenge on day 0. Depletion was confirmed by immunohistochemistry on day ^+^10 relative to challenge day 0. Panel 1 (A and B): indicating CD169 tagged with BV421, F4/80 tagged with Alexa Fluor-594, CD11c tagged with Alexa Fluor, CD3 tagged with Alexa Fluor-660 and overlayed image of a *Py*17X CAF01 vaccinated mouse that received (**A**) clodronate liposomes or (**B**) control PBS liposomes, respectively. Panel 2 (C and D): indicating CD209b tagged with Alexa Fluor-488, DAPI, and overlayed image of *Py*17X-DDA/TDB vaccinated mouse that received (**C**) clodronate liposomes or (**D**) control PBS liposomes, respectively.
